# Enhanced Yield of Pepper Plants Promoted by Soil Application of Volatiles From Cell-Free Fungal Culture Filtrates Is Associated With Activation of the Beneficial Soil Microbiota

**DOI:** 10.3389/fpls.2021.752653

**Published:** 2021-10-21

**Authors:** Edurne Baroja-Fernández, Goizeder Almagro, Ángela María Sánchez-López, Abdellatif Bahaji, Samuel Gámez-Arcas, Nuria De Diego, Karel Dolezal, Francisco José Muñoz, Eric Climent Sanz, Javier Pozueta-Romero

**Affiliations:** ^1^Instituto de Agrobiotecnología (CSIC/Gobierno de Navarra), Nafarroa, Spain; ^2^Centre of the Region Haná for Biotechnological and Agricultural Research, Czech Advanced Technology and Research Institute, Olomouc, Czechia; ^3^Department of Chemical Biology, Faculty of Science, Palacký University Olomouc, Olomouc, Czechia; ^4^Laboratory of Growth Regulators, Institute of Experimental Botany of the Czech Academy of Sciences, Faculty of Science, Palacký University Olomouc, Olomouc, Czechia; ^5^ADM Biopolis, Valencia, Spain; ^6^Instituto de Hortofruticultura Subtropical y Mediterránea “La Mayora” (IHSM-UMA-CSIC) Campus de Teatinos, Málaga, Spain

**Keywords:** biostimulant, fruit yield, fungal phytopathogen, plant growth promoting microorganism, plant-microbe interaction, soil microbiota, volatile organic compounds

## Abstract

Plants communicate with microorganisms by exchanging chemical signals throughout the phytosphere. Such interactions are important not only for plant productivity and fitness, but also for terrestrial ecosystem functioning. It is known that beneficial microorganisms emit diffusible substances including volatile organic compounds (VOCs) that promote growth. Consistently, soil application of cell-free culture filtrates (CF) of beneficial soil and plant-associated microorganisms enhances plant growth and yield. However, how this treatment acts in plants and whether it alters the resident soil microbiota, are largely unknown. In this work we characterized the responses of pepper (*Capsicum annuum* L.) plants cultured under both greenhouse and open field conditions and of soil microbiota to soil application of CFs of beneficial and phytopathogenic fungi. To evaluate the contribution of VOCs occurring in the CFs to these responses, we characterized the responses of plants and of soil microbiota to application of distillates (DE) of the fungal CFs. CFs and their respective DEs contained the same potentially biogenic VOCs, and application of these extracts enhanced root growth and fruit yield, and altered the nutritional characteristics of fruits. High-throughput amplicon sequencing of bacterial 16S and fungal ITS rRNA genes of the soil microbiota revealed that the CF and DE treatments altered the microbial community compositions, and led to strong enrichment of the populations of the same beneficial bacterial and fungal taxa. Our findings show that CFs of both beneficial and phytopathogenic fungi can be used as biostimulants, and provide evidence that VOCs occurring in the fungal CFs act as mediators of the plants’ responses to soil application of fungal CFs through stimulation of the beneficial soil microbiota.

## Introduction

Plants are metaorganisms that host a complex and dynamic consortium of bacteria, fungi, archaea and protists that communicate with plants by exchanging chemical signals throughout the rhizosphere and endosphere. Such interactions are important not only for plant productivity and fitness, but also for terrestrial ecosystem functioning (Philippot et al., 2013; De-la-Peña and Loyola-Vargas, 2014; Delgado-Baquerizo et al., 2016; Huang et al., 2019; Yu et al., 2019; Prudent et al., 2020). Therefore, understanding the relationships between plant growth and soil microbial populations is necessary when seeking new and efficient ecological intensification and agricultural strategies based on manipulation of the soil microbiota. A safe and environmentally friendly approach to increase crop yield and/or protect plants from abiotic stress and pests while reducing the use of agrochemicals is based on the inoculation of the soil with plant growth promoting microorganisms that act as biostimulants (López-Bucio et al., 2015; Ahmad et al., 2018). These microorganisms emit diffusible substances including phytohormones and amino acids that promote root branching and nutrient uptake, enhance photosynthesis, alter metabolism, confer resistance to abiotic stresses and pathogens (Badri et al., 2013a; De-la-Peña and Loyola-Vargas, 2014; López-Bucio et al., 2015), and stimulate resident beneficial microbial communities in the phytosphere (Kröber et al., 2014; Fiorentino et al., 2018), thereby boosting plant growth and yield. Consistently, agronomic studies have shown that the application to soil of cell-free filtrates of cultures of plant-associated beneficial microbes enhances seed germination, seedling growth and crop yield (Aldesuquy et al., 1998; Varma et al., 1999; Bagde et al., 2011; Sung et al., 2011; Rahman et al., 2012; Yandigeri et al., 2012; Kaur et al., 2019).

Microorganisms also emit a plethora of volatile compounds (VCs) with molecular masses of less than 300 Da that act as efficient semiochemicals in interkingdom communication, participating in countless interactions among plants and microorganisms, and exerting a strong effect on microbial communities (Schmidt et al., 2016; Werner et al., 2016; Schulz-Bohm et al., 2017). VCs emitted by beneficial microorganisms promote growth and developmental changes, enhance photosynthesis, improve nutrient acquisition, elicit plant defenses and inhibit the growth of plant pathogens when applied via the air (Ryu et al., 2003; Chaurasia et al., 2005; Kai et al., 2007; Zhang et al., 2008, 2009; Gutiérrez-Luna et al., 2010; Kottb et al., 2015; Garnica-Vergara et al., 2016; Sánchez-López et al., 2016; Guo et al., 2020). Recent studies have shown that this capacity also extends to VCs emitted by phytopathogens and microorganisms that do not normally interact mutualistically with plants (Ezquer et al., 2010; Bitas et al., 2015; Sánchez-López et al., 2016; Cordovez et al., 2017; Li et al., 2018; García-Gómez et al., 2019, 2020; Moisan et al., 2019), although several lines of evidence indicate that the mechanisms involved in some plants’ responses to VCs emitted by beneficial and pathogenic microorganisms are different (Hernández-Calderón et al., 2018; García-Gómez et al., 2020). In *Arabidopsis*, enhanced growth and photosynthesis promoted by air application of fungal VCs is associated with increases in levels of active forms of cytokinins (CKs), photosynthetic pigments and transitory starch in leaves, together with reductions in abscisic acid (ABA) contents, and changes in the transcriptome and proteome through mechanisms involving signaling of redox-activated photosynthesis and long-distance communication between roots and the aerial part of the plant (Zhang et al., 2008; Sánchez-López et al., 2016; Ameztoy et al., 2019, 2021; García-Gómez et al., 2020).

To date, studies on the response of plants to soil application of cell-free filtrates from cultures of beneficial microorganisms have been centered on the effect of these compounds on plant growth and yield, but have not explored their modes/mechanisms of action in plants and/or their effects on resident soil microbial communities (Aldesuquy et al., 1998; Bagde et al., 2011; Sung et al., 2011; Rahman et al., 2012; Yandigeri et al., 2012; Kaur et al., 2019). Using the beneficial fungus *Trichoderma harzianum* and two fungal phytopathogens (i.e., *Alternaria alternata* and *Penicillium aurantiogriseum*) here we conducted studies to address the question of whether soil application of cell-free filtrates of beneficial and pathogenic fungal cultures can improve growth and yield of a plant species of agronomic interest (i.e., pepper [*Capsicum annuum* L.]) cultured under greenhouse and open field conditions, and whether this response is mediated by mechanisms comparable to those triggered by air application of fungal VCs. In addition, using amplicon sequencing targeting of the fungal ITS and bacterial 16S rRNA genes, we investigated whether filtrates of fungal cultures are capable of altering the indigenous soil microbial communities. Furthermore, using distillates (DEs) obtained from the fungal cultures, we evaluated the contribution made by fungal organic VCs (VOCs) in these responses. Our findings show that soil application of cell-free filtrates of beneficial and pathogenic fungi promotes root growth and enhances yield of pepper plants at least partially through mechanisms wherein activation of beneficial soil microbiota promoted by VOCs occurring in the fungal culture filtrates (CFs) could play important roles.

## Materials and Methods

### Preparation of Filtrates and Distillates From Fungal Cultures

*Trichoderma harzianum* (CECT 2413), *A. alternata* (CECT 20912) and *P. aurantiogriseum* (CECT 20226) from glycerol stocks were inoculated into 500 mL Erlenmeyer flasks containing 200 mL liquid Murashige and Skoog (MS) medium supplemented with 90 mM sucrose. Cultures were incubated at 28°C with constant shaking at 180 rpm on a rotatory shaker for 7 days. Next, 100 mL of the cultures were poured into 2 L Erlenmeyer flasks containing 1 L MS-sucrose medium and incubated at 28°C with constant shaking at 180 rpm for 3 days, after which the fungal mycelium was removed using Whatman #1 filter paper. The resulting filtrate was sterilized using a 0.22 μm Millipore membrane filter and stored at 4°C whilst awaiting further use. DEs were obtained by distilling the CFs at 50°C using a R3000 (BUCHI) rotavapor following manufacturer’s instructions^1^.

### Plants, Growth Conditions, Soil Application of Fungal Culture Filtrates and Distillates and Sampling

This work was carried out using Sweet Italian and Piquillo pepper plants cultured under greenhouse and field conditions, respectively. Seeds were sown and cultured in the dark at 24°C on filter paper moistened with distilled water. Ten days after sowing, the pepper seedlings were transplanted into small pots (250 mL) filled with peat:sand:vermiculite mixture (1:1:1) and cultured for 4 weeks in growth chambers providing 16 h light (90 μmol photons sec^–1^ m^–2^), 25°C /8 h dark, 15°C cycles. For greenhouse trials, plants were transplanted to 6 L pots filled with peat:sand:vermiculite mixture (1:1:1) and cultured for 7 weeks with natural light under 16 h 25°C /8 h 15°C conditions. Treatments with fungal CFs and DEs started when the plants developed their first two true leaves. Increasing volumes (1.5, 3, 6, 10, 15, 20, and 25 mL per plant) of CFs and DEs were sequentially applied on soil once a week over 7 weeks. Each treatment was applied to twelve plants. Controls for DE-treated plants were plants irrigated with distilled water. Controls for CF-treated plants were plants irrigated with diluted (1:3) Murashige and Skoog (MS) medium supplemented with 1.5 mM each of glucose and fructose, which is the approximate concentration of both sugars in the three CFs. At the selected sampling times, leaves and commercial fruits were harvested and immediately ground to a fine powder in liquid nitrogen, then stored at −80°C prior to further biochemical characterization. “Commercial” Sweet Italian pepper fruits are defined as those having a minimum length of 14 cm.

Field trials were conducted at the INTIA’s experimental station located near Sartaguda (Navarre, Spain) between May and September of 2016 and 2018. Plants were transplanted from 250 mL pots (see above) to 22.40 m^2^ plots. Each plot contained 70 plants. Each treatment was applied to plants in three randomly distributed plots. The distance between plants in the same row was 35 cm and the distance between rows was 160 cm. Using a water meter (Contazara, CZ3000RI), plants were drip-irrigated with a 1:25 dilution of the original CF extract (10,4 L/plot) twice: one month after transplanting and at flowering. Using the same method, control plants were irrigated with a 1:25 dilution of the MS medium supplemented with 1.5 mM each of glucose and fructose stock (10.4 L/plot). Commercial fruits were harvested on three occasions: first, third and fourth week of September. Those destined for compositional analyses were immediately ground to a fine powder in liquid nitrogen and stored at −80°C. “Commercial” Piquillo pepper fruits are defined as those having 9–10 cm length, 40–45 cm width, 40–45 g weight and 1.5–3 mm pericarp thickness.

### Analytical Procedures

Soluble sugars in CFs and frozen powders of leaves and fruits were determined by HPLC with pulsed amperometric detection on a IC3000 Dionex system, as described in Bahaji et al. (2015a). Contents of amino acids in CFs, leaves and fruits were measured by HPLC according to Loiret et al. (2009). Levels of CKs, ABA and indole acetic acid (IAA) in CFs and leaves were determined following Novák et al. (2008), Pěnčík et al. (2009), and Floková et al. (2014), respectively. Starch content in leaves was measured using an amyloglucosidase–based test kit (Boehringer Mannheim, Germany). Recovery experiments were carried out by adding known amounts of metabolite standards to the samples immediately after the addition of extraction solutions. The difference between the measurements from samples with and without added standards was used as an estimate of the percentage recovery. All presented concentrations of metabolites were corrected for losses during extraction.

Volatile organic compounds in CFs and DEs were determined by gas chromatography-mass spectrometry (GC-MS) using the solid-phase microextraction (SPME) technique as described by García-Gómez et al. (2019). Briefly, 5 mL of CFs and DEs were transferred into a 10 mL headspace vial (Agilent ref. 5183–4475) containing a magnetic stir bar. After 30 min of equilibration at 50°C with shaking, VOCs were adsorbed for 30 min using solid phase microextraction fiber coated with DVB/CAR/PDMS. The fiber was injected into an Agilent 7890A gas chromatograph containing a 30 m × 0.25 mm fused silica HP-5MS column. Mass spectral analyses were carried out with an Agilent 5975C instrument. Mass spectra of VOCs were compared to those obtained from the NIST library and identifications were confirmed using commercially available standard compounds.

### Fruit Texture Analyses

Fruit hardness was determined using a penetrometer (FT-327 Penetrometer, PAMPOLS), by measuring the depth or rate of penetration of a rod driven into the fruit with a known force.

### Determinations of Photosynthetic Parameters

Gas exchange rates were determined at 25°C with a photosynthetic photon flux density of 350 μmol m^–2^ s^–1^, as described by Bahaji et al. (2015b) using a portable LI-COR 6400 gas exchange photosynthesis system (LI-COR, Lincoln, NE, United States). Net rates of CO_2_ assimilation (*A*_*n*_) and stomatal conductance (*g*_*s*_) were calculated as described by von Caemmerer and Farquhar (1981). Intrinsic water use efficiency (WUE*i*) was calculated as the ratio of *A*_*n*_ to *g*_*s*_ as described by Flexas et al. (2016). Chlorophyll contents in leaves were determined using a portable chlorophyll meter (SPAD-502 Minolta, Japan).

### Soil Sample Preparation, DNA Extraction and High-Throughput Sequencing

Soil microbiome studies were conducted at ADM-Biopolis (Paterna, Valencia, Spain) using soil from the 250 mL pots with plants cultured for 4 weeks in growth chambers (see above). Three days after the third treatment with fungal CFs or DEs, three replicate samples each consisting of all the soil from three pots were collected, mixed, sieved to a < 2 mm particle size, lyophilized and stored at −80°C. Soil DNA was extracted from 10 grams of soil of each replicate using a DNeasy Mericon kit from QIAGEN. The quantity and purity of DNA were examined with a NanoDrop ND-1000 spectrophotometer (NanoDrop Technologies, Delaware, United States). The bacterial 16S rRNA and fungal ITS libraries were constructed following the “16S metagenomics sequencing library preparation” protocol given for the Illumina MiSeq system, and using optimized primers targeting the hypervariable V3 and V4 regions of the 16S rRNA gene for the bacterial profile (Klindworth et al., 2013) and primers that cover ITS1-ITS2 for the fungal profile (Op De Beeck et al., 2014). Libraries were sequenced on an Illumina MiSeq instrument and uploaded to SRA archive with reference PRJNA748689. PCR primers present in the raw sequences were trimmed using cutadapt v2.6 (Martin, 2011), and merged with BBMap v38.26. Sequences were also filtered with BBMap in order to remove those with low quality or shorter than 200 nucleotides. Chimeras were filtered with the UCHIME algorithm in the USEARCH package (Edgar et al., 2011) and high-quality sequences were clustered into operational taxonomic units (OTUs), using cd-hit v4.8.1 (Fu et al., 2012) with a similarity threshold of 99% (Abarenkov et al., 2010). A total of 5,282,314 bacterial sequence reads were obtained from 24 samples. The numbers of high-quality sequences per sample varied from 98,030 to 140,027 and were grouped into 6,241 OTUs. In addition, a total of 3,283,782 fungal sequence reads were obtained. The number of high-quality sequences per sample varied from 50,007 to 90,123, resulting in 1,567 OTUs. Rarefaction curves obtained using R vegan package^2^ suggested that sequencing depth was sufficient to cover most of the detectable bacterial and fungal OTUs as the curves began to plateau or reached their asymptote for all sample types by treatment (Supplementary Figure 1). OTUs were annotated against NCBI nt database using blast tool and keeping up to the 10 best hits, in order to differentiate species whose 16S region is unique from species that share the sequenced 16S region with closes organisms. This way, species mentioned in this report were those that could be fully identified. Control soils for DE- and CF-treated soils were those irrigated with water and diluted MS medium supplemented with 1.5 mM each of glucose and fructose, respectively.

### Data Analyses

The significance of differences in growth, yield, number of leaves and fruits per plant, photosynthesis and metabolite contents between CF- and DE-treated and non-treated plants was statistically evaluated with Student’s *t*-test using SPSS software. Differences were considered significant if *P* < 0.05. Both microbiome α- and β-diversity statistical analyses were performed using the R package “Phyloseq” v1.23.0 (De Mendiburu and Simon, 2015) on log-normalized data in order to avoid an increase in error rates due to rarefaction. In particular, α-diversity was measured using Shannon, Simpson and Richness indexes, and β-diversity was studied using Bray-curtis distance and PCoA. ANOVA test was used to check the differences in α-diversity, and PERMANOVA test was used with β-diversity contrasts. All differential abundance contrasts were done with DESeq2 package (Love et al., 2014) and validated using Wald text.

## Results

### Effects of Soil Application of Fungal Cell-Free Culture Filtrates on Plant Growth and Yield

**FIGURE 1:**
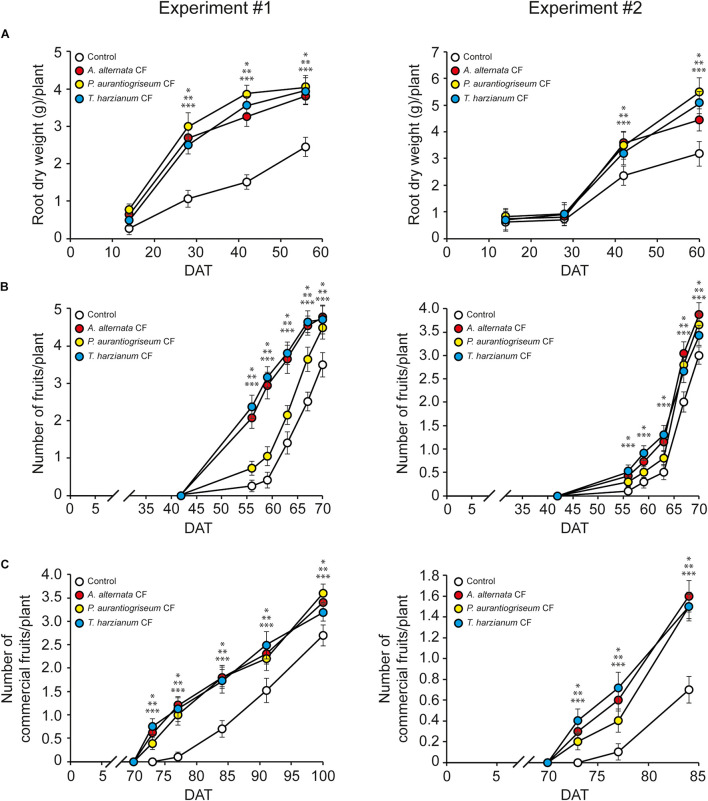
Soil application of fungal CFs stimulates root growth and induces early fruit development in pepper plants. Data over the time for root dry weight **(A)** and number of total fruits **(B)** and commercial fruits **(C)** per pepper plant (cv. Sweet Italian) after the first soil application of *A. alternata, P. aurantiogriseum*, and *T. harzianum* CFs. Plants were grown under greenhouse conditions. Values were obtained from two independent experiments and represent the mean ± SD of 12 different plants. Asterisks indicate significant differences between CF-treated plants and plants irrigated with CF control according to a Student’s *t*-test (*P < 0.05, + *A. alternata* CF; **P < 0.05, + *P. aurantiogriseum* CF; ***P < 0.05, + *T. harzianum* CF). DAT, Days after treatment.

We characterized growth and fruit yield responses of pepper plants (cv. Sweet Italian) grown under greenhouse conditions to soil application of CFs from *T. harzianum, A. alternata* and *P. aurantiogriseum*. In two independent experiments, the three CFs stimulated root growth (Figure 1A; Supplementary Figure 2), induced fruit development (Figures 1B,C) and enhanced the number of commercial fruits per plant and yield (Table 1). Furthermore, these treatments induced the accumulation of free amino acids in fruits. The most prominent ones were Ser, the long-distance nitrogen transport amino acids Asp, Glu, Asn and Gln and the stress-responsive GABA (Table 2). Soil application of the three CFs did not alter plant height, number of leaves per plant, stem thickness (Supplementary Figure 3) or fruit size and weight (Table 1). Soil application of *A. alternata* CFs, but not *P. aurantiogriseum* and *T. harzianum* CFs, enhanced ABA content in leaves (Supplementary Table 1). In addition, *P. aurantiogriseum* and *T. harzianum* CFs, but not *A. alternata* CFs, enhanced active CK contents in leaves (Supplementary Table 1). Moreover, soil application of the three fungal CFs did not affect leaf chlorophyll content, *A*_*n*_ and *g*_*s*_ in leaves (Supplementary Figure 4). Consistently, WUE*i* and levels of starch and soluble sugars, regarded here as primary photosynthates, were not affected by the CF treatment (Supplementary Figure 4).

**TABLE 1:** Soil application of *A. alternata, P. aurantiogriseum*, and *T. harzianum* CFs enhances commercial fruit number and yield of pepper plants (cv. Sweet Italian) cultured under greenhouse conditions.

	Experiment #1				Experiment #2			
		
Treatment	Yield (g/plant)	Number of fruits per plant	Fruit length (cm)	Fruit weight (g)	Yield (g/plant)	Number of fruits per plant	Fruit length (cm)	Fruit weight (g)
Control	180 ± 21	7.0 ± 0.4	12.8 ± 0.6	25.8 ± 4.2	198 ± 31	7.5 ± 0.7	12.9 ± 0.7	26.5 ± 5.6
+ *A. alternata* CF	366 ± 23*	16.0 ± 0.9*	13.0 ± 1.0	22.9 ± 3.8	347 ± 13*	11.5 ± 1.0*	12.7 ± 1.1	30.2 ± 6.4
+ *T. harzianum* CF	369 ± 22*	16.8 ± 0.9*	13.2 ± 0.7	22.0 ± 3.5	295 ± 17*	11.0 ± 0.9*	13.4 ± 1.7	26.8 ± 5.3
+ *P. aurantiogriseum* CF	381 ± 11*	15.0 ± 0.7*	13.7 ± 0.6	25.4 ± 3.6	309 ± 21*	12.0 ± 1.1*	13.2 ± 1.0	35.8 ± 7.6

*The results were obtained from two independent experiments and are the mean ± SE of twelve different plants per treatment. Asterisks indicate statistically significant difference in the CF treatments versus the controls according to a Student’s *t*-test (*P* < 0.05).*

**TABLE 2:** Free amino acids and sugar contents (expressed as nmol g^–1^ DW) in commercial fruits of pepper plants (cv. Sweet Italian) cultured under greenhouse conditions with or without drench application of fungal CFs.

		Control	+ *A. alternata* CF	+ *P. aurantiogriseum* CF	+ *T. harzianum* CF
Amino acids	Asp	115.4 ± 5.1	133.0 ± 5.0*	140.3 ± 3.1*	137.5 ± 4.8*
	Glu	93.4 ± 4.3	104.8 ± 4.0*	105.6 ± 2.0*	103.2 ± 3.8*
	Asn	78.2 ± 2.5	89.3 ± 2.6*	94.7 ± 2.2*	87.9 ± 3.1*
	Gln	129.4 ± 1.6	185.5 ± 4.7*	143.7 ± 2.9*	145.8 ± 4.5*
	Ser	58.5 ± 7.6	90.7 ± 8.5*	74.2 ± 7.6*	78.4 ± 7.0*
	GABA	117.4 ± 2.7	143.3 ± 3.8*	145.4 ± 9.6*	148.0 ± 7.1*
	Gly	52.1 ± 3.7	63.0 ± 3.3*	59.2 ± 2.0	56.6 ± 2.8
	His	54.1 ± 3.8	62.9 ± 3.5	60.5 ± 1.8	57.6 ± 2.8
	Thr	90.2 ± 4.8	106.6 ± 4.6*	101.6 ± 2.1	97.5 ± 4.1
	Ala	484.2 ± 14.2	516.4 ± 15.3	538.9 ± 12.8*	501.0 ± 27.1
	Arg	71.0 ± 5.1	87.0 ± 4.6*	79.3 ± 2.4	76.7 ± 3.7
	Pro	43.0 ± 7.6	37.7 ± 2.7	34.7 ± 3.1	39.6 ± 3.3
	Tyr	91.7 ± 6.8	106.5 ± 6.2	102.3 ± 3.3	97.3 ± 5.0
	Val	40.7 ± 0.9	47.6 ± 1.1*	46.4 ± 0.9*	44.7 ± 1.4
	Met	58.4 ± 4.5	67.8 ± 4.1	56.5 ± 2.1	62.0 ± 3.2
	Ile	66.7 ± 4.0	77.0 ± 3.8	73.6 ± 2.0	70.8 ± 3.2
	Leu	66.5 ± 4.6	77.6 ± 4.1	74.0 ± 2.0	71.0 ± 3.3
	Lys	4.2 ± 1.0	4.6 ± 1.1	4.1 ± 1.3	4.4 ± 1.2
	Phe	77.7 ± 5.5	90.7 ± 5.0*	86.9 ± 2.7*	85.9 ± 3.1*
Sugars	Glucose	90,600 ± 4,190	87,640 ± 2,560	95,330 ± 4,340	91,480 ± 3,410
	Fructose	56,710 ± 2,080	5,836 ± 2,300	63,370 ± 2,680	60,870 ± 2,460
	Sucrose	1,050 ± 60	1,140 ± 80	1,070 ± 30	1,106 ± 61

*Values represent the mean ± SE from two independent experiments each consisting of three biological replicates corresponding to a pool of fruits from twelve plants. Asterisks indicate statistically significant difference in the CF treatments versus the controls according to a Student’s *t*-test (*P* < 0.05).*

We also characterized the responses of pepper plants (cv. Piquillo) grown in the field to soil application of the three fungal CFs. In two independent experiments, these treatments enhanced the number of commercial fruits per plant and yield (Table 3) and altered fruit composition. As shown in Table 4, the CFs induced the accumulation of sucrose and long-distance nitrogen transport amino acids in fruits. CF treatments did not exert any significant effect on agronomic traits such as fruit size and weight (Table 3) and pericarp texture and thickness (Supplementary Figure 5).

**TABLE 3:** Soil application of *A. alternata, P. aurantiogriseum*, and *T. harzianum* CFs enhances commercial fruit number and yield of pepper plants (cv. Piquillo) cultured under open field conditions.

	Experiment #1				Experiment #2			
		
Treatment	Yield (Kg/ha)	Number of fruits per ha (×1,000)	Fruit length (cm)	Fruit weight (g)	Yield (Kg/ha)	Number of fruits per ha (×1,000)	Fruit length (cm)	Fruit weight (g)
Control	27,480 ± 1,457	631 ± 32	8.4 ± 0.1	43.5 ± 1.2	23,103 ± 1,745	510 ± 28	8.7 ± 0.2	45.3 ± 1.9
+ *A. alternata* CF	33,192 ± 1,610*	739 ± 34*	8.6 ± 0.1	44.9 ± 1.1	29,742 ± 1,962*	658 ± 33*	8.5 ± 0.1	45.2 ± 1.5
+ *T. harzianum* CF	31,134 ± 1,576*	699 ± 34*	8.5 ± 0.1	44.5 ± 1.2	29,388 ± 1,767*	632 ± 39*	8.7 ± 0.2	46.5 ± 1.5
+ *P. aurantiogriseum* CF	36,055 ± 1,601*	828 ± 39*	8.5 ± 0.1	43.5 ± 1.2	29,315 ± 1,918*	612 ± 34*	8.8 ± 0.2	47.9 ± 2.0

*The results are the mean ± SE of 210 plants per treatment. Experiment #1 and #2 were conducted in 2016 and 2018, respectively. Asterisks indicate statistically significant difference in the CF treatments versus the control according to a Student’s *t*-test (*P* < 0.05).*

**TABLE 4:** Free amino acids and sugar contents (expressed as nmol g^–1^ DW) in commercial fruits of pepper plants (cv. Piquillo) cultured under open field conditions with or without drench application of fungal CFs.

		Control	+ *A. alternata* CF	+ *P. aurantiogriseum* CF	+ *T. harzianum* CF
Amino acids	Asp	1,798 ± 347	2,920 ± 373*	2,488 ± 192*	2,874 ± 383*
	Glu	814 ± 113	1,040 ± 69*	1,047 ± 46*	1,162 ± 83*
	Asn	31,981 ± 1,023	48,151 ± 2,085*	34,167 ± 548*	44,147 ± 3,208*
	Gln	1,758 ± 396	2,161 ± 457	1,558 ± 184	2,443 ± 398
	Ser	2,476 ± 438	3,044 ± 546	2,980 ± 274	3,430 ± 668
	GABA	823 ± 116	915 ± 115	928 ± 46	892 ± 75
	Gly	291 ± 48	273 ± 25	563 ± 30*	320 ± 29
	His	260 ± 22	282 ± 25	375 ± 26*	504 ± 41
	Thr	1,429 ± 268	1,552 ± 169	1,466 ± 114	1,587 ± 225
	Ala	6,956 ± 1,568	6,183 ± 1,144	10,317 ± 639	7,485 ± 1,547
	Arg	369 ± 58	370 ± 43	359 ± 36	393 ± 30
	Pro	2,275 ± 271	3,313 ± 384	602 ± 102*	2,215 ± 568
	Tyr	455 ± 80	423 ± 82	393 ± 27	456 ± 46
	Val	916 ± 140	803 ± 112	879 ± 57	952 ± 87
	Met	293 ± 43	286 ± 51	200 ± 13*	298 ± 33
	Ile	426 ± 71	410 ± 64	360 ± 25	436 ± 37
	Leu	398 ± 58	376 ± 61	328 ± 13	371 ± 36
	Lys	254 ± 48	244 ± 28	259 ± 47	246 ± 12
	Phe	650 ± 37	778 ± 37*	734 ± 24*	735 ± 33*
Sugars	Glucose	47,001 ± 213	47,822 ± 787	44,817 ± 216	44,513 ± 1,997
	Fructose	83,530 ± 1,473	87,711 ± 1,472	77,606 ± 4,228	73,513 ± 2,907
	Sucrose	11,602 ± 340	15,610 ± 562*	12,890 ± 338*	13,509 ± 434*

*Values represent the mean ± SE for two independent experiments each consisting of three biological replicates corresponding to a pool of fruits from five plants randomly distributed in three plots. Asterisks indicate statistically significant difference in the CF treatments versus the controls according to Student’s *t*-test (*P* < 0.05).*

### Compositional Analysis of the Fungal Cell-Free Culture Filtrates

Culture filtrates of the three fungal species contained complex mixtures of amino acids (Supplementary Table 2). Some of these amino acids (e.g., Glu, GABA, Pro, and Ala) are known to be involved in signaling mechanisms for environmental changes that confer stress resistance to plants and/or affect their growth and development (Forde, 2014; Podlešáková et al., 2019). The hormones of the three CFs showed contrasting differences. *P. aurantiogriseum* CFs contained very much higher levels of CKs than those of *A. alternata* and *T. harzianum* (Supplementary Table 3). In addition, *P. aurantiogriseum* CFs contained higher levels of IAA (16,188 ± 239 pmol/L) than those of *A. alternata* and *T. harzianum* (1,049 ± 23 and 1,475 ± 530 pmol/L, respectively). Moreover, ABA content in *T. harzianum* CF (20.1 ± 5.5 pmol/L) was higher than in *A. alternata* and *P. aurantiogriseum* CFs (7.4 ± 1.6 and 7.2 ± 1.6, respectively). Analyses of the organic volatilomes of the three CFs identified a total of 80 VOCs (Table 5). Although the volatilomes were highly different in each of the CFs, 13 compounds (i.e., ethanol, 1-propanol, 1-butanol-3-methyl, benzaldehyde-2-methyl, 2,4-dimethyl-1-heptene, 2-phenylethyl alcohol, benzene-1,3-bis(1,1-dimethylethyl), acetic acid, propanoic acid ethyl ester, butyrolactone, 2-heptanone-4-methyl, 2-heptanone-4,6-dimethyl and 2-nonanone) could be identified in all three (Table 5). No sucrose could be detected in the three CFs, but all these extracts contained 0.5–1.5 mM each of glucose and fructose, likely due to strong extracellular fungal sucrolytic activity.

**TABLE 5:** Headspace analysis (SPME/GC-MS) of VOCs collected from filtrates and DEs obtained from *A. alternata, P. aurantiogriseum*, and *T. harzianum* liquid cultures.

Retention time (min)	Chemical family	Compound	*A. alternata*		*P. aurantiogriseum*		*T. harzianum*	
					
			CF	DE	CF	DE	CF	DE
	Alcohol							
1.47		ethanol^a^	+	+	+	+	+	+
1.82		1-propanol^a^	+	+	+	−	+	+
2.26		1-propanol, 2 methyl	+	+	−	−	+	+
2.59		1-butanol^a^	+	+	−	−	−	−
3.64		1-butanol, 3-methyl^a,b^	+	+	+	+	+	+
3.68		1-butanol, 2-methyl^b^	+	+	−	−	+	+
4.22		1-pentanol	+	−	−	−	−	−
5.15		2-butanol, 2-methyl, acetate	+	+	+	+	−	−
6.08		3-methyl-1-pentanol	−	−	−	−	+	+
6.61		1-propanol, 3-ethoxy	−	−	+	−	−	−
6.71		1-hexanol^a,b^	+	−	−	−	−	−
6.92		3-hexanol, 4-methyl	+	−	−	−	−	−
6.95		1-butanol, 3- methyl-, acetate	−	−	−	−	+	+
7.01		1-butanol, 2- methyl-, acetate	−	−	−	−	+	+
7.44		2-hexanol, 2,5-dimethyl	+	+	−	−	−	−
7.61		2-heptanol	−	−	−	−	+	+
11.52		1-hexanol, 2-ethyl	+	+	+	+	−	−
13.71		2-nonanol	−	−	−	−	+	+
19.43		2-undecanol	−	−	−	−	+	−
	Aldehyde							
9.38		benzaldehyde^a,b^	−	−	+	−	−	−
13.08		benzaldehyde, 2-methyl	+	+	+	+	+	+
	Alkane							
2.17		propane, 2-ethoxy-2-methyl	+	+	−	−	−	−
2.96		2,3-pentanedione	+	−	−	−	−	−
4.57		2,3-hexanedione	+	−	−	−	−	−
5.40		heptane, 2,4-dimethyl	+	+	−	−	−	−
	Alkene							
1.95		1-pentene, 2-methyl	+	+	−	−	−	−
5.91		2,4-dimethyl-1-heptene	+	+	+	+	+	+
	Aromatic compound							
4.15		toluene^a^	−	−	+	+	+	+
6.7		p-xylene	+	+	+	+	−	−
7.28		styrene^a^	+	+	+	−	−	−
11.95		benzeneacetaldehyde	−	−	+	+	−	−
14.21		phenylethyl alcohol^b^	+	+	+	+	+	+
15.63		phenol, 4-ethyl	−	−	−	−	+	+
17.90		benzeneacetic acid, ethyl ester	−	−	+	−	−	−
18.04		2-acetyl-3-methylthiophene	−	−	−	−	+	+
18.16		benzene, 1,3-bis(1,1-dimethylethyl)	+	+	+	+	+	+
23.76		2,5-di-tert-butyl-1,4-benzoquinone	+	+	−	−	+	+
	Carboxylic acid							
1.92		ethyl acetate^a^	−	−	−	−	+	−
3.11		acetic acid^a,b^	+	+	+	+	+	+
3.20		propanoic acid, ethyl ester	+	−	+	−	+	+
4.04		propanoic acid, 2-methyl, ethyl ester	−	−	+	−	+	−
4.35		isobutyl acetate	+	+	−	−	+	+
4.70		propanoic acid, 2-methyl	−	−	+	−	−	−
4.95		butanoic acid, ethyl ester	−	−	−	−	+	−
5.07		propanoic acid^a^	−	−	+	−	−	−
5.16		propanoic acid, propyl ester	−	−	−	−	+	−
6.19		butanoic acid, 2-methyl- ethyl ester	−	−	−	−	+	−
6.57		butanoic acid, 3-methyl	−	−	+	−	−	−
	Ketone							
2.84		2-pentanone	−	−	−	−	+	−
4.42		acetoin^a,b^	+	+	−	−	−	−
6.19		2,3-butanediol^a,b^	−	−	+	−	−	−
7.32		2-heptanone^b^	−	−	−	−	+	−
8.29		butyrolactone	+	−	+	+	+	+
8.75		2-heptanone, 4-methyl	+	+	+	+	+	+
10.34		2-heptanone, 4,6-dimethyl	+	+	+	+	+	+
12.31		2-nonanone^a,b^	+	+	+	+	+	+
12.64		acetophenone^b^	−	−	+	+	−	−
	Monoterpene							
9.89		2-methylenebornane	−	−	+	−	−	−
15.82		(+)-isomenthol	−	−	−	−	+	+
15.84		2-methylisoborneol^a^	−	−	+	−	−	−
17.41		citronellol	−	−	−	−	+	+
18.62		citral	−	−	−	−	+	−
	Sesquiterpene							
22.16		β-cedrene	−	−	+	+	−	−
22.51		γ-elemene	−	−	+	−	−	−
22.91		*cis-*thujopsene^a,b^	+	+	+	+	−	−
23.16		unknown sesq.	−	−	−	−	+	+
23.25		α-longipinene^a,b^	+	+	−	−	−	−
23.28		α-cadinene	+	+	−	−	−	−
23.51		cuparene^a^	+	+	−	−	−	−
23.48		*cis-*β-farnesene	−	−	−	−	+	−
24.08		β-chamigrene	+	−	−	−	−	−
24.17		α-himachalene	+	+	+	−	−	−
24.63		cedrene-V6	+	+	−	−	−	−
24.71		α-chamigrene^a^	+	+	+	−	−	−
25.15		β-humulene	−	−	−	−	+	−
26.02		α-bisabolol	−	−	−	−	+	+
26.99		widdrol	+	+	−	−	−	−
27.33		β-selinene	−	−	−	−	+	+
27.51		α-gurjunene	+	+	−	−	+	−
27.67		α-cedrene^a^	−	−	−	−	+	−

*^*a*^Compounds identified by comparison of RT and mass spectral data to those of authentic compounds. Other compounds were identified by comparing their mass spectral data to spectra from the NIST library and by comparing their linear retention indices (using an n-alkane scale) to literature values. ^*b*^Compounds previously reported to affect plant growth (Ryu et al., 2003; Ditengou et al., 2015; Kim et al., 2017; Camarena-Pozos et al., 2019; Jiang et al., 2019; Allen and Allen, 2020). −, not detected.*

### Soil Application of Fungal Distillates Stimulates Root Growth and Enhances Fruit Yield of Pepper Plants

**FIGURE 2:**
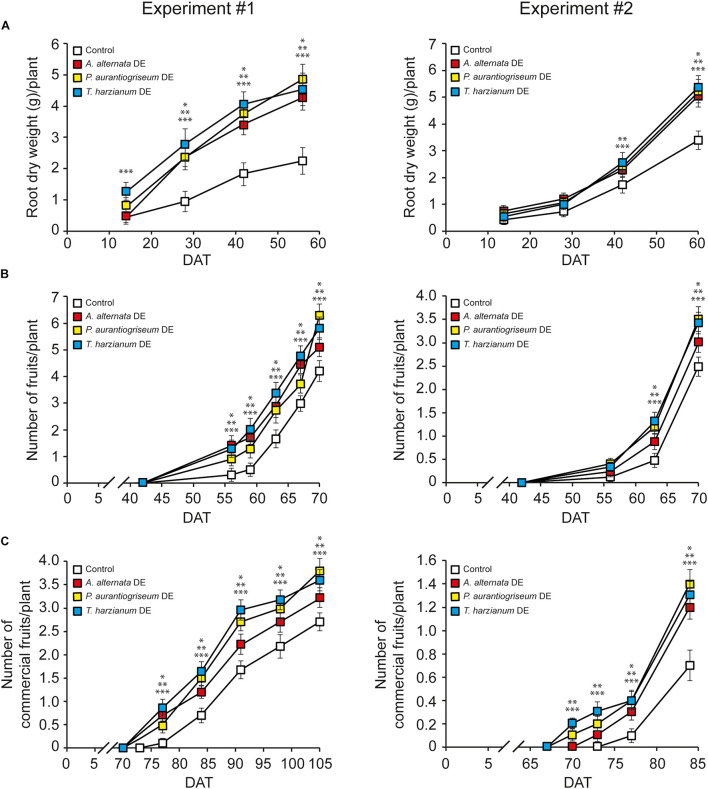
Soil application of fungal DEs stimulates root growth and induces early fruit development in pepper plants. Data over the time for root dry weight **(A)** and number of total fruits **(B)** and commercial fruits **(C)** per pepper plant (cv. Sweet Italian) after the first application of *A. alternata, P. aurantiogriseum*, and *T. harzianum* DEs on soil. Plants were grown under greenhouse conditions. Values were obtained from two independent experiments and represent the mean ± SD of 12 different plants. Asterisks indicate significant differences between DE-treated plants and plants irrigated with DE control according to a Student’s *t*-test (*P < 0.05, + *A. alternata* DE; **P < 0.05, + *P. aurantiogriseum* DE; ***P < 0.05, + *T. harzianum* DE). DAT, Days after treatment.

We next investigated the contribution of VOCs to the responses of pepper plants to CFs. To this end, we characterized growth and yield responses of Sweet Italian pepper plants grown under greenhouse conditions to soil application of DEs obtained from cultures of the three fungal species. We reasoned that if the plant’s responses to CFs were solely due to non-volatile bioactive compounds such as hormones and amino acids, DEs should trigger at most a weak response. Conversely, if VOCs have high action potentials, DEs should still trigger strong responses in plants. As a first step in these studies, we conducted compositional analyses of the VOCs in the DEs. These analyses revealed that ca. 75% of the VOCs identified in the CFs were also identified in the DEs (Table 5). The responses of pepper plants to DEs were comparable to those of CF-treated plants as the three DEs stimulated root growth (Figure 2A; Supplementary Figure 2), induced fruit production (Figures 2B,C), and increased the number of commercial fruits per plant and yield (Table 6). DEs did not significantly affect fruit size (Table 6). The overall data indicate that VOCs are important determinants of pepper plants’ responses to soil application of fungal CFs.

**TABLE 6:** Soil application of DEs obtained from *A. alternata, P. aurantiogriseum*, and *T. harzianum* cultures enhances commercial fruit number and yield of pepper plants (cv. Sweet Italian) cultured under greenhouse conditions.

	Experiment #1				Experiment #2			
		
Treatment	Yield (g/plant)	Number of fruits per plant	Fruit length (cm)	Fruit weight (g)	Yield (g/plant)	Number of fruits per plant	Fruit length (cm)	Fruit weight (g)
Water	162 ± 12	6.5 ± 0.4	12.9 ± 0.5	25.0 ± 1.7	150 ± 15	5.9 ± 0.5	13.3 ± 0.9	25.5 ± 1.3
+ *A. alternata* DE	343 ± 23*	14.0 ± 1.0*	14.0 ± 0.9	24.5 ± 1.9	275 ± 18*	11.0 ± 1.1*	13.2 ± 0.6	25.0 ± 1.7
+ *T. harzianum* DE	329 ± 19*	13.8 ± 0.9*	13.7 ± 1.0	23.9 ± 1.8	270 ± 20*	10.0 ± 1.0*	13.5 ± 1.1	27.0 ± 3.1
+ *P. aurantiogriseum* DE	355 ± 21*	13.1 ± 0.9*	13.5 ± 0.7	27.1 ± 2.0	290 ± 22*	11.5 ± 1.2*	13.1 ± 0.9	25.2 ± 3.6

*The results were obtained from two independent experiments and are the mean ± SE of twelve different plants per treatment. Asterisks indicate statistically significant difference in the DE treatments versus the controls according to a Student’s *t*-test (*P* < 0.05).*

### Impact of Soil Application of Fungal Culture Filtrates and Distillates on the Indigenous Soil Microbial Abundance, Diversity and Composition

We next investigated the impact of soil application of the filtrates and DEs obtained from cultures of the three fungal species on the composition of the indigenous soil bacterial and fungal communities.

**FIGURE 3:**
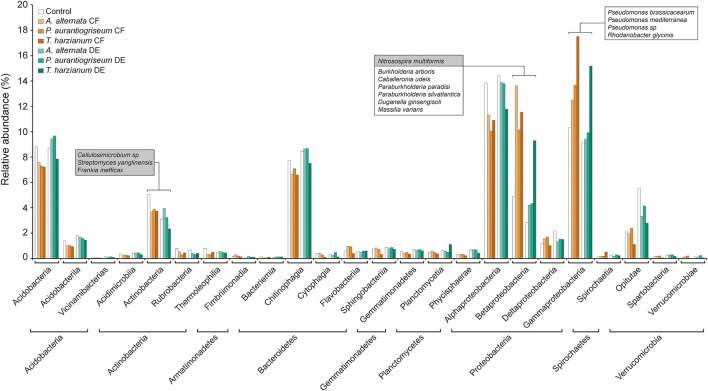
Soil application of fungal CFs and DEs alters the composition of the soil bacterial microbiota. The figure shows the relative abundances of bacteria in soils irrigated with fungal CFs (highlighted in brown) and DEs (highlighted in green). Relative abundances of bacteria in control soils are shown in white bars. Bacteria were arranged according to their phylum and class. Species discussed here whose relative abundances are significantly altered by the CF and DE treatments are boxed. Names of species enriched and impoverished by soil application of CFs and DEs are included in white and gray boxes, respectively. Data obtained from Supplementary Table 4.

**FIGURE 4:**
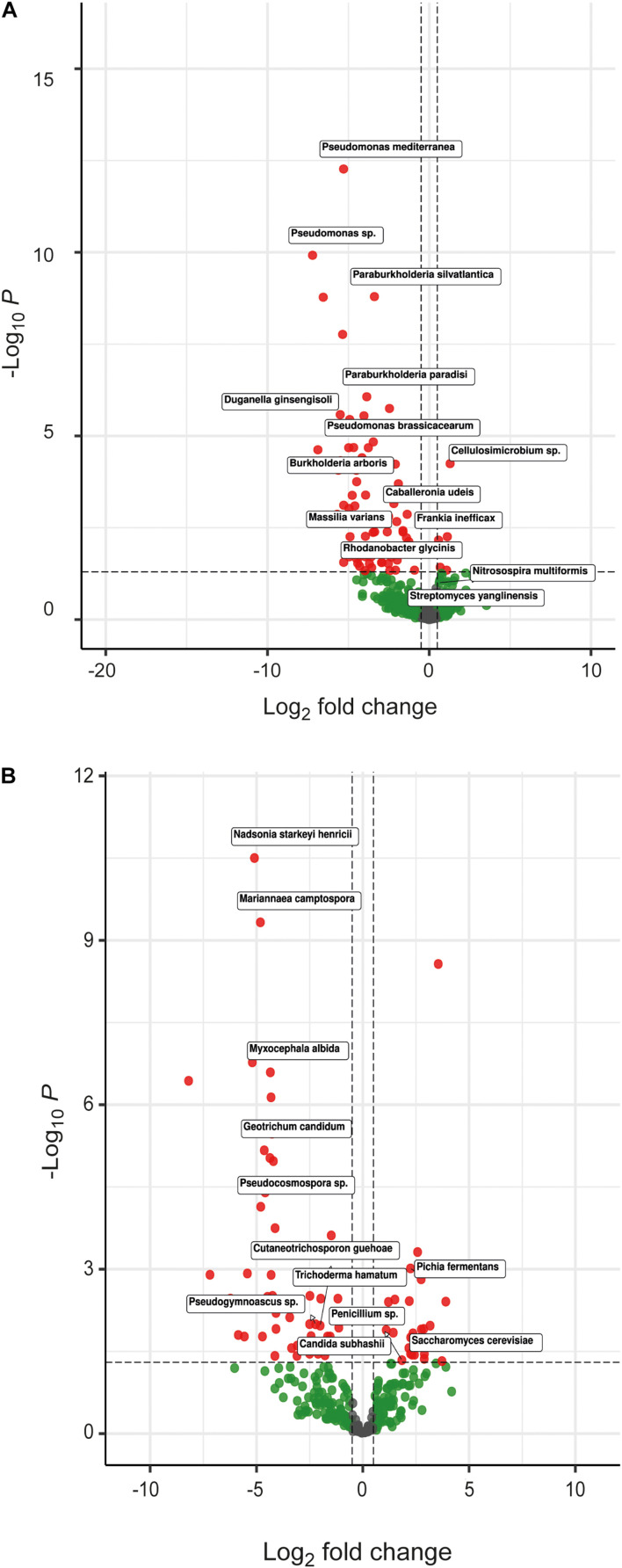
Volcano plot of the differential abundances of microbial profiles at species level, based on DESeq2 results. This plot shows the **(A)** bacterial and **(B)** fungal species that were significantly up- and down-regulated by soil application of fungal CFs and DEs (negative and positive log2FC, respectively). Species discussed here are boxed. Thresholds of the volcano plot were established at 0.5 log2Foldchange and 0.05 adjusted *p*-value (red dots).

**FIGURE 5:**
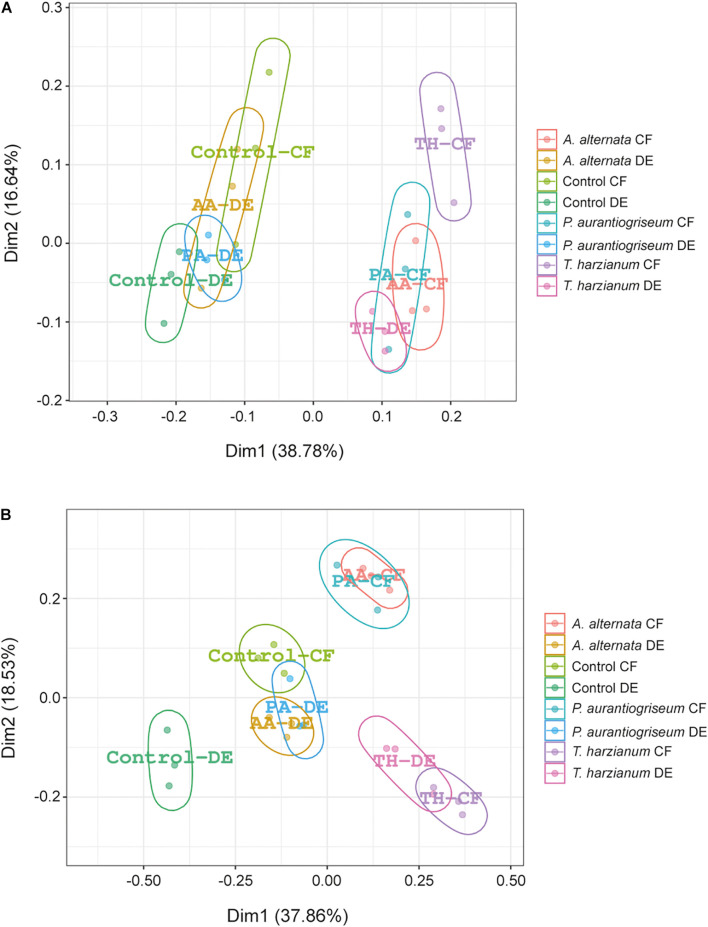
Effect of soil application of fungal CFs and DEs on microbial diversity in soil. The graphics represent the results of principal coordinates analyses (PCoA) showing the β-diversities of the **(A)** bacterial and **(B)** fungal communities in soils treated with CFs and DEs of *A. alternata* (AA-CF and AA-DE, respectively), *P. aurantiogriseum* (PA-CF and PA-DE, respectively) and *T. harzianum* (TH-CF and TH-DE, respectively) and control soils.

**FIGURE 6:**
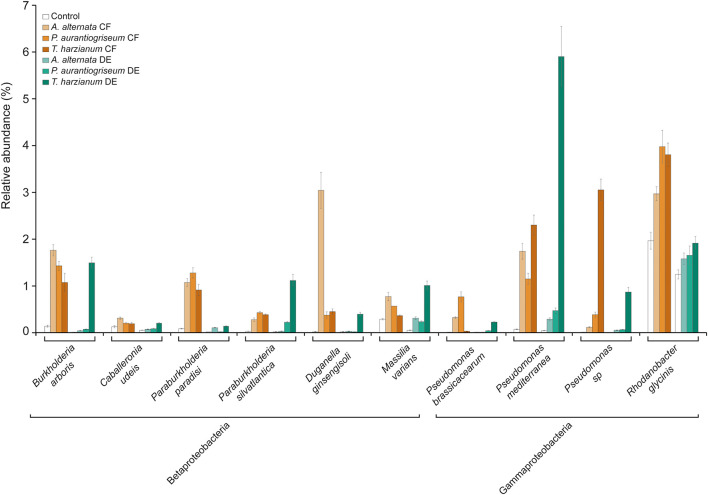
Soil application of fungal CFs and DEs strongly enriches the same beneficial soil bacterial species. The figure shows the relative abundances of bacterial species in soil discussed here that were significantly up-regulated by soil application of fungal CFs (highlighted in brown) and DEs (highlighted in green) according to a Student’s *t*-test (*P* < 0.05). Values represent the mean ± SD. Relative abundances of bacteria in control soils are shown in white bars. Data obtained from Supplementary Table 4.

The bacterial microbiome profile based on amplicon sequencing of the bacterial 16S rRNA gene is shown in Supplementary Tables 4, 5. Bacterial abundances in CF- and DE-treated soils were similar to those of control soils (Supplementary Table 6). At class level, most OTUs were assigned to *Acidobacteria, Actinobacteria, Chitinophagia, Alphaproteobacteria, Betaproteobacteria*, and *Gammaproteobacteria*, representing ca. 90% of all assigned sequences (Supplementary Table 4; Figure 3). Results of the DESeq2 differential abundance test suggest that the application of the three CFs and DEs significantly altered the soil bacterial community composition (Supplementary Table 5). The most obvious change caused by the treatments was the increase in the relative abundance of *Betaproteobacteria* (Supplementary Table 4; Figure 3). At genus level, the three CF and DE treatments enriched populations of *Pseudomonas* (*P. brassicacearum* and *P. mediterranea*), *Paraburkholderia* (*P. paradisi* and *P. silvatlantica*), *Burkholderia* (*B. arboris*), *Rhodoferax, Rhodanobacter* (*R. glycinis*), and *Massilia* (*M. varians*), and reduced populations of *Cellulosimicrobium* (*C. funkei*) (Figures 3, 4A). Soil application of the fungal CFs and DEs did not alter the number of species, as measured by the Richness index (Supplementary Figure 6A). At species level, the *T. harzianum* CFs and DEs reduced α-bacterial diversity, as measured by the Shannon and Simpson indexes (Supplementary Figure 6A). At the same level, the 3 CFs and the DE from *T. harzianum* altered the bacterial β-diversity, as shown in the Bray-curtis PCoA plot (Figure 5A), indicating that these extracts have strong capacity to alter the bacterial community composition in soil. In general, the different CF and DE treatments strongly enriched the populations of *Caballeronia udeis* and *Duganella ginsengisoli* (Supplementary Table 4; Figures 3, 4A, 6), and impoverished those of *Streptomyces yanglinensis, Frankia inefficax*, and *Nitrosospira multiformis* (Supplementary Table 4; Figures 3, 4A).

**FIGURE 7:**
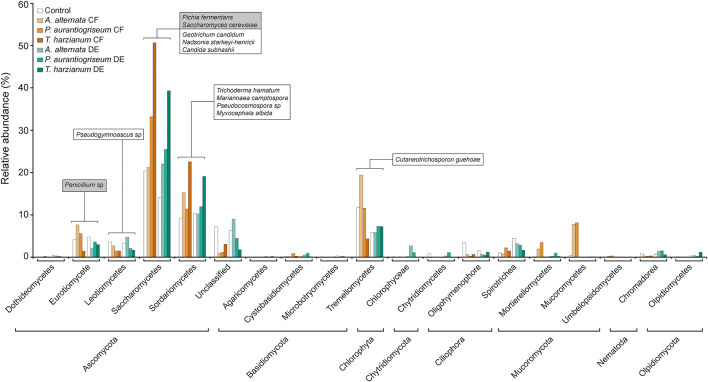
Soil application of fungal CFs and DEs alters the composition of the soil fungal microbiota. The figure shows the relative abundances of fungi in soils irrigated with fungal CFs (highlighted in brown) and DEs (highlighted in green). Relative abundances of fungi in control soils are shown in white bars. Fungi were arranged according to their phylum and class. Species discussed here whose relative abundances are significantly altered by the CF and DE treatments are boxed. Names of species enriched and impoverished by soil application of CFs and DEs are included in white and gray boxes, respectively. Data obtained from Supplementary Table 4.

**FIGURE 8:**
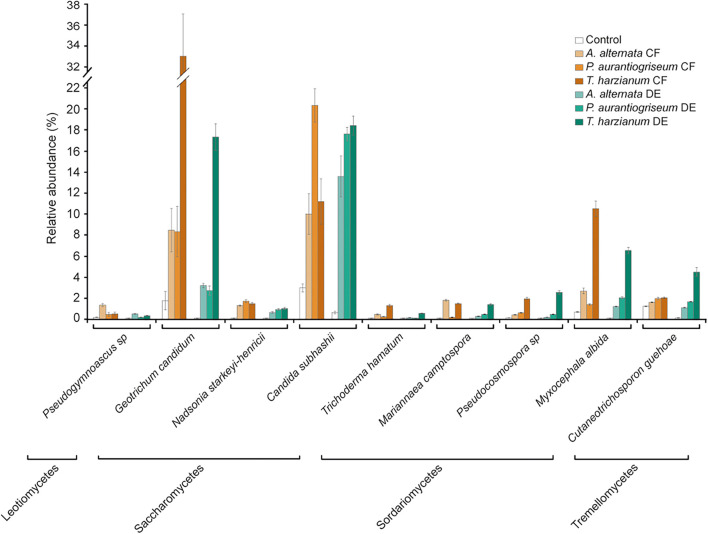
Soil application of fungal CFs and DEs strongly enriches the same beneficial soil fungal species. The figure shows the relative abundances of fungal species in soil discussed here that were significantly up-regulated by soil application of fungal CFs (highlighted in brown) and DEs (highlighted in green) according to a Student’s *t*-test (*P* < 0.05). Values represent the mean ± SD. Relative abundances of fungi in control soils are shown in white bars. Data obtained from Supplementary Table 4.

The fungal microbiome profile based on amplicon sequencing of the fungal ITS1-ITS2 gene is shown in Supplementary Tables 4, 7. Fungal abundances in CF- and DE-treated soils were similar to those of control soils (Supplementary Table 6). At class level, the dominant taxa were Saccharomycetes, Sordariomycetes and Tremellomycetes, representing 30–90% of all assigned sequences (Supplementary Table 4; Figure 7). Results of the DESeq2 differential abundance test suggest that the application of the three CFs and DEs significantly altered the soil fungal community composition (Supplementary Table 7). The largest shifts caused by the CFs and DEs treatments in the fungal microbiota were the increases of the relative abundances of Mucoromycetes, Saccharomycetes and Sordariomycetes (Supplementary Table 4; Figure 7). At genus level, CF and DE treatments enriched populations of *Geotrichum* (*G. candidum*), *Candida* (*C. subhashii*), *Trichoderma* (*T. hamatum*) and *Myxocephala* (*M. albida*), and reduced those of *Saccharomyces* (*S. cerevisiae*) (Figure 4B). At species level, the CF, but not DE treatments altered the fungal α-diversity of the samples (Supplementary Figure 6B). However, the three CF and DE treatments significantly altered β-diversity, as shown in the Bray-curtis PCoA plot (Figure 5B). The CF and the DE treatments strongly enriched the populations of *Pseudogymnoascus* sp., *Nadsonia starkeyi-henricii, Mariannaea camptospora, Pseudocosmospora rogersonii* and *Myxocephala albida*, (Supplementary Table 4; Figures 7, 8), and reduced those of *Penicillium sp.* and *Pichia fermentans* (Supplementary Table 4; Figures 4B, 7).

## Discussion

### Culture Filtrates of Both Beneficial and Phytopathogenic Fungi Can Be Used as Biostimulants to Improve Crop Yield

Here, we showed that soil application of CFs of beneficial and pathogenic fungi promotes root growth, accelerates fruit development, and increases the yield of commercial fruits of pepper plants cultured under greenhouse and field conditions. The differences observed between non-induced plants of different independent experiments could be ascribed to non-controlled environmental factors, such as differences in temperature and light intensity and quality. Therefore, CFs of both beneficial and phytopathogenic fungi can be used as biostimulants to improve crop yield. Because CFs alter fruit composition, they can potentially be used to improve fruit quality.

Root growth stimulation and enhanced fruit yield promoted by soil application of *A. alternata* and *P. aurantiogriseum* CFs was surprising as it is well known that many fungal phytopathogens release secondary metabolites and proteins in their culture media that display phytotoxicity in tests on tissue culture germinating seeds, plantlets, calli and cellular suspensions (McLean, 1996; Lou et al., 2013; Rao et al., 2014; Ogórek, 2016; Parveen et al., 2019). Phytotoxin formation by microbes is sensitive to a number of diverse environmental factors, including the microbial culture medium composition and the culturing duration and conditions (e.g., temperature, light and aeration) (Berestetskiy, 2008). Here, we report data obtained using filtrates extracted from *A. alternata* and *P. aurantiogriseum* grown for 3 days on MS-sucrose, a minimal medium normally used for *in vitro* culture of plants, but not for microbiology studies. Filtrates of 7 day-old cultures exert a negative effect on plant growth (not shown). Therefore, the contrasting differences between our results and those of previous studies can be ascribed to the fact that, under the experimental conditions used in this study, *A. alternata* and *P. aurantiogriseum* are not active with respect to phytotoxin production.

### Volatile Organic Compounds Are Strong Determinants of Pepper Plants’ Responses to Soil Application of Fungal Culture Filtrates

Here, we showed that the vast majority of VOCs released by fungi to aqueous culture media can be “rescued” by distillation, and analyzed the effect of soil application of these compounds in plants. This is, to our knowledge, the first study of the response of plants to soil application of VOCs distilled from microbial cultures. Under greenhouse conditions, the responses of pepper plants to soil application of the three DEs were comparable to those of CF-treated plants. Thus, in our experimental system, VOCs appear to be strong determinants of the plants’ responses to fungal CFs. Unlike air VC application, soil DE application did not promote shoot growth and photosynthesis, did not enhance WUEi and the contents of photosynthetic pigments, starch and soluble sugars in leaves, and did not reduce leaf ABA content, indicating that the mechanisms involved in the responses of plants to soil and air applications of volatiles are different.

Some potentially relevant bioactive VOCs present in at least one of the CFs are 1-butanol-3-methyl, 1-butanol-2-methyl, 1-hexanol, benzaldehyde, 2-phenylethyl alcohol, acetic acid, acetoin, 2,3-butanediol, 2-heptanone, 2-nonanone, acetophenone, *cis-*thujopsene and α-longipinene, as they have been shown to promote shoot growth and root developmental changes when exogenously supplied to plants (Ryu et al., 2003; Ditengou et al., 2015; Kanchiswamy et al., 2015; Fincheira et al., 2017; Kim et al., 2017; Camarena-Pozos et al., 2019; Jiang et al., 2019; Allen and Allen, 2020). Some of these compounds (i.e., 1-butanol-3-methyl, 2-phenylethyl alcohol, acetic acid, and 2-nonanone) were present in the three DEs used in this study. It is likely that these compounds play important roles in the response of pepper plants to soil application of fungal DEs.

Distillates from fungal cultures used in this study are free from bioactive compounds such as phytohormones and amino acids. We must emphasize that the data obtained in this study do not imply that components of the CFs other than VOCs are not involved in the response of pepper plants to soil application of CFs. Soil application of amino acids and hormones affects growth and yield of plants and they are the main constituents of some biostimulants (Sudadi, 2012; Calvo et al., 2014; du Jardin, 2015; Koprna et al., 2016; Khan et al., 2019). On the other hand, microbially produced amino acids and phytohormones such as CKs, ABA and auxins are determinants of plant growth and yield (Arkhipova et al., 2005; Spaepen et al., 2007; Contreras-Cornejo et al., 2009; Ortíz-Castro et al., 2009; Zahir et al., 2010; Radhakrishnan et al., 2014; Chanclud and Morel, 2016). Because CFs of the three fungal species contain CK, ABA, IAA and amino acids, it is conceivable that the response of pepper plants to soil application of fungal CFs is due not only to VOCs, but also to the actions of fungal hormones and/or amino acids. The specific contributions of either amino acids and hormones of the CFs on the plant response to the CFs remains to be tested.

### Soil Application of Fungal Culture Filtrates Increases the Relative Abundance of Resident Beneficial Soil Microbiota at Least Partially Through Mechanisms Involving Volatile Organic Compound Action

To our knowledge, this is the first report showing that soil application of fungal CFs and VOCs leads to restructuring of the soil microbiota. These treatments altered the bacterial and fungal β-diversities in soil, and enhanced the relative abundances of several taxa of the dominant soil bacterial classes Betaproteobacteria and Gammaproteobacteria and those of the fungal classes Saccharomycetes and Sordariomycetes. In addition, soil applications of CFs and DEs altered the abundance of the same bacterial and fungal species. Therefore, in our experimental system, VOCs appear to be important mediators in the response of the soil microbiota to fungal CFs. It is well known that soil and foliar application of non-volatile phytohormones and amino acid-enriched extracts promotes changes in the composition of soil and plant microbiota (Lebeis et al., 2015; Colla et al., 2017; Wang et al., 2019). Because CFs of the three fungal species used in this study contain amino acids and hormones, it is conceivable that the response of the soil microbiota to soil application of fungal CFs is also due to the action of these compounds. This is consistent with the fact that CFs promote stronger changes in bacterial β-diversity than DEs.

It has recently been proposed that benefits derived from some biostimulants might be due to shifts in the composition of the plant-associated and soil microbial communities (Mahnert et al., 2018; Luziatelli et al., 2019; Renaut et al., 2019; Wang et al., 2019). Here, we found that soil application of the three fungal CFs and DEs enriches the populations of the same bacterial species. Some of them (e.g., *P. silvatlantica, C. udeis, P. mediterranea, P. brassicacearum*, and *R. glycinis*) have been shown to act as plant growth promoters that solubilize mineral phosphate, fix nitrogen, produce siderophores, synthesize hormones (e.g., IAA and CKs) and/or antagonize microbial phytopathogens (Perin et al., 2006; Belimov et al., 2007; Kalita and Małek, 2017; Rabhi et al., 2018; Deng et al., 2019; Lee et al., 2019; Mannaa et al., 2019; Gu et al., 2020). In addition, all CF and DE treatments strongly enriched the populations of *C. subhashii*, a yeast antagonist of filamentous fungal phytopathogens (Hilber-Bodmer et al., 2017), and that of *G. candidum.* Although the latter fungus is known to be the causal agent of decays of numerous fruits (Palou et al., 2009; Talibi et al., 2012), it has been shown to promote growth when inoculated in plants (Waqas et al., 2017) probably due to its capacity to synthesize IAA, solubilize mineral phosphate and produce VOCs that inhibit microbial phytopathogens (Wu et al., 2012; Fu et al., 2016; George et al., 2019; Chen et al., 2020). It is worth noting that the relative abundances of most of the above microbial species were very low in the microbial populations of non-treated soils and became very high after the CF and DE treatments. In the case of *G. candidum*, for instance, relative abundances increased from 0.06 to 1.78% in non-treated soils to up to ca. 20 and 35% in DE- and CF-treated soils, respectively. *C. subhashii* relative abundances increased from 0.6 to 18.4% after fungal DE treatments whereas those of *P. mediterranea* increased from 0.04% to 0.07% in non-treated soils to up to 5.9 and 2.3% in DE- and CF-treated soils, respectively. Therefore, we propose that enhanced root growth and yield of pepper plants and changes in the fruit composition promoted by fungal CFs and DEs are at least partly due to strong activation of the beneficial soil microbiota. It remains to be determined if any of the bacterial or fungal species that are enriched in the rhizosphere upon application of the biostimulants may have a dominant effect over the microbial population, modulating root growth, architecture and biomass partitioning for fruit yield.

Ten compounds (i.e., ethanol, 1-butanol-3-methyl, benzaldehyde-2-methyl, 2,4-dimethyl-1-heptene, 2-phenylethyl alcohol, benzene-1,3-bis(1,1-dimethylethyl), acetic acid, 2-heptanone-4-methyl, 2-heptanone-4,6-dimethyl and 2-nonanone) are present in the three fungal DEs. It is likely that, individually or collectively, these compounds play important roles in the VOC-mediated activation of the beneficial microbiota in CF-treated soils. It is noteworthy that the response of the soil microbial community to *T. harzianum* DEs was stronger than those of soils treated with *A. alternata* and *P. aurantiogriseum* DEs, indicating that *T. harzianum* DEs contain higher levels of bioactive VOCs than those of *A. alternata* and *P. aurantiogriseum* and/or different proportions of the same VOCs. Additionally, and/or alternatively, it is possible that *T. harzianum* DEs contain VOCs with strong action potential that are not present in *A. alternata* and *P. aurantiogriseum* DEs. Such compounds could include 3-methyl-1-pentanol; 1-butanol, 3- methyl-, acetate; 1-butanol, 2- methyl-, acetate; 2-heptanol; 2-nonanol; phenol, 4 ethyl; 2-acetyl-3-methylthiophene; (+)-isomenthol; citronellol; α-bisabolol and β-selinene. It is evident that further efforts will be necessary to characterize the action potentials of these compounds on beneficial microbial populations.

### Concluding and Additional Remarks

Results presented in this work show that CFs of both beneficial and phytopathogenic fungi can be used as biostimulants, and provide evidence that VOCs occurring in the fungal CFs act as mediators of the plants’ responses to soil application of fungal CFs through stimulation of the beneficial soil microbiota. Data provided here should motivate the applications of translational biology needed to reinforce agricultural practices with more natural, cheaper and sustainable approaches.

The discovery that soil application of VOCs from both beneficial and pathogenic microorganisms can enhance root growth and yield and promote similar changes in the soil microbial communities extends knowledge of the diversity and complexity of the interactions involved in modulation of the physiology of the plant and its interaction with surrounding microbes, raising questions regarding the evolution of the processes, their ecological significance and potential applications. Because microbes respond to microbial VOCs (Schmidt et al., 2016; Werner et al., 2016; Schulz-Bohm et al., 2017), we propose that enrichment of the soil beneficial microbiota promoted by application of fungal VOCs is due, at least partly, to direct action of these compounds on the microorganisms. It is well known that roots have the ability to influence the rhizosphere microbiota by releasing metabolites including amino acids, flavonoids, terpenes, organic acids, phenolic compounds, VOCs, etc. (Badri et al., 2013b; Schulz-Bohm et al., 2017; Sasse et al., 2018; Huang et al., 2019). Air application of microbial VCs enhances root growth (Gutiérrez-Luna et al., 2010; Ditengou et al., 2015; García-Gómez et al., 2019) and ethylene production (García-Gómez et al., 2020), and promotes changes in root exudate composition (Hernández-Calderón et al., 2018). Recent studies have shown that ethylene emitted by roots can control the rhizosphere microbial community assembly, indicating that this volatile hormone can be used by plants as a cue to interact with soil microorganisms (Chen et al., 2020). Therefore, it is likely that VOC-promoted changes of the soil microbiota are also due to enhanced root growth, altered composition of exudates and enhanced ethylene emissions by roots. Clearly, further experiments are necessary to test these hypotheses.

Microbes and their volatiles contribute in the various aspects of biotic stress management. The release of volatiles by beneficial microbes directly protects plants by inhibiting growth of pathogenic organisms, and indirectly elicit plant defense response by enhancing immune response against pathogen attack (De-la-Peña and Loyola-Vargas, 2014; Kanchiswamy et al., 2015). It has been suggested that phytopathogens or their constituents may provide opportunities for plant production or be useful for specific biotechnological applications (Tarkowski and Vereecke, 2014). It remains to be determined if soil application of CFs and DEs from fungal phytopathogens cross-protect plants from other pathogens.

## One Sentence Summary

We show that soil application of cell-free filtrates of beneficial and pathogenic fungi promotes root growth and enhances yield of pepper plants through mechanisms wherein activation of beneficial soil microbiota by VOCs occurring in the fungal culture filtrates could play important roles.

## Data Availability Statement

The original contributions presented in the study are publicly available. This data can be found here: National Center for Biotechnology Information (NCBI) BioProject database under accession number PRJNA748689.

## Author Contributions

EB-F and JP-R designed the experiments, analyzed the data, and wrote the article with contributions from all authors. EB-F, GA, ÁS-L, AB, SG-A, ND, KD, and FJM performed most of the experiments. ECS performed the microbiome statistical analyses. JP-R supervised the experiments and conceived the project and research plans. All authors contributed to the article and approved the submitted version.

## Conflict of Interest

The authors declare that the research was conducted in the absence of any commercial or financial relationships that could be construed as a potential conflict of interest.

## Publisher’s Note

All claims expressed in this article are solely those of the authors and do not necessarily represent those of their affiliated organizations, or those of the publisher, the editors and the reviewers. Any product that may be evaluated in this article, or claim that may be made by its manufacturer, is not guaranteed or endorsed by the publisher.

## Footnotes

1
www.manualslib.com/manual/1589626/Buchi-Rotavapor-R-3000.html

2
https://cran.r-project.org/web/packages/vegan

## Abbreviations

*A*_*n*_: Net rates of CO_2_ assimilation
CFs: culture filtrates
CKs: cytokinins
DEs: distillates
MS: Murashige and Skoog
OTUs: operational taxonomic units
SPME: solid-phase microextraction
*g*_*s*_: stomatal conductance
VCs: volatile compounds
VOCs: organic VCs
WUE*i*: intrinsic water use efficiency.
