# Supplementary material for: Enhanced Yield of Pepper Plants Promoted by Soil Application of Volatiles From Cell-Free Fungal Culture Filtrates Is Associated With Activation of the Beneficial Soil Microbiota
Source: Front Plant Sci. 2021 Oct 21;12:752653. doi: 10.3389/fpls.2021.752653 (PMC8566893; doi:10.3389/fpls.2021.752653)
Supplement: Supplementary file 1 [file Data_Sheet_1.zip › Supplementary Figures 1-6.PDF]

A

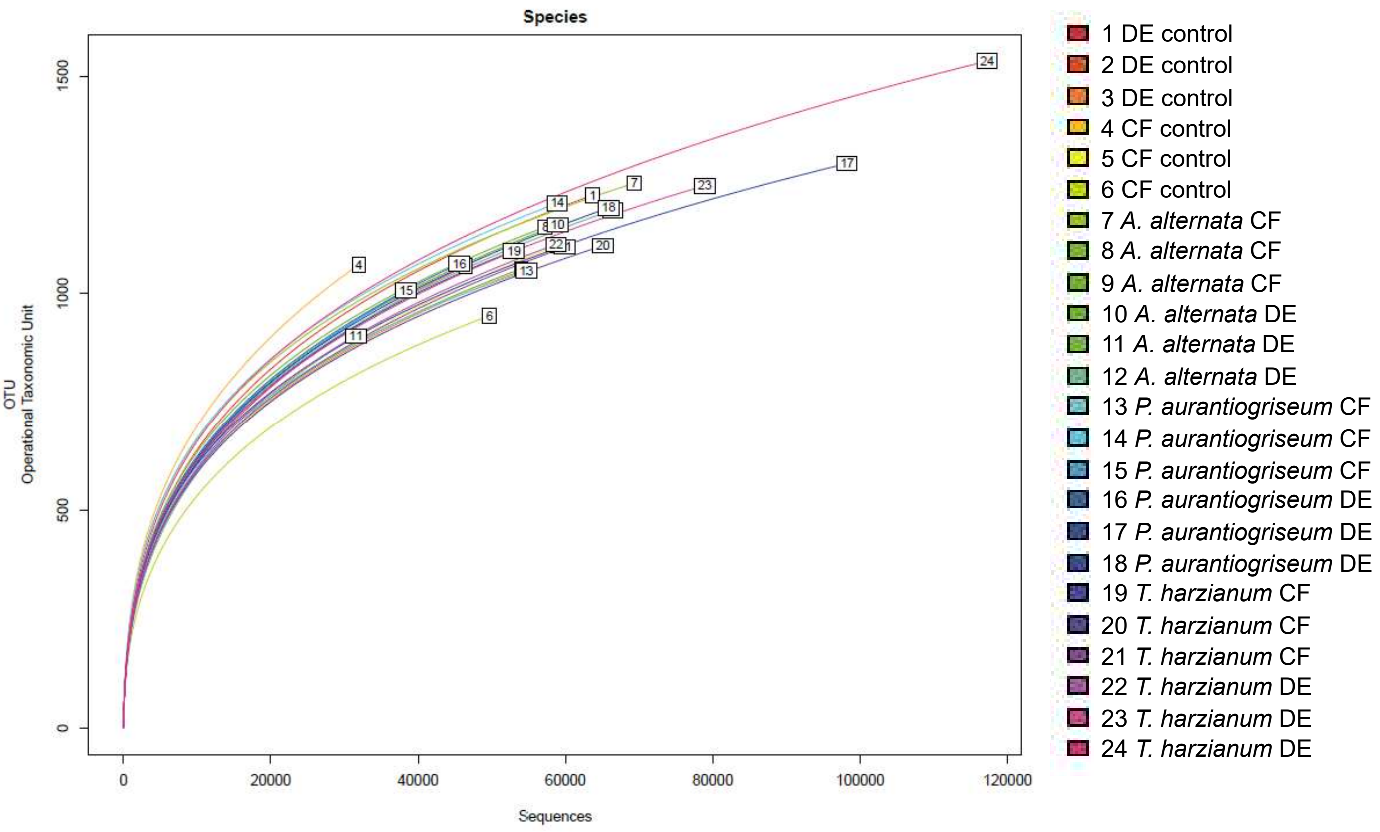

B

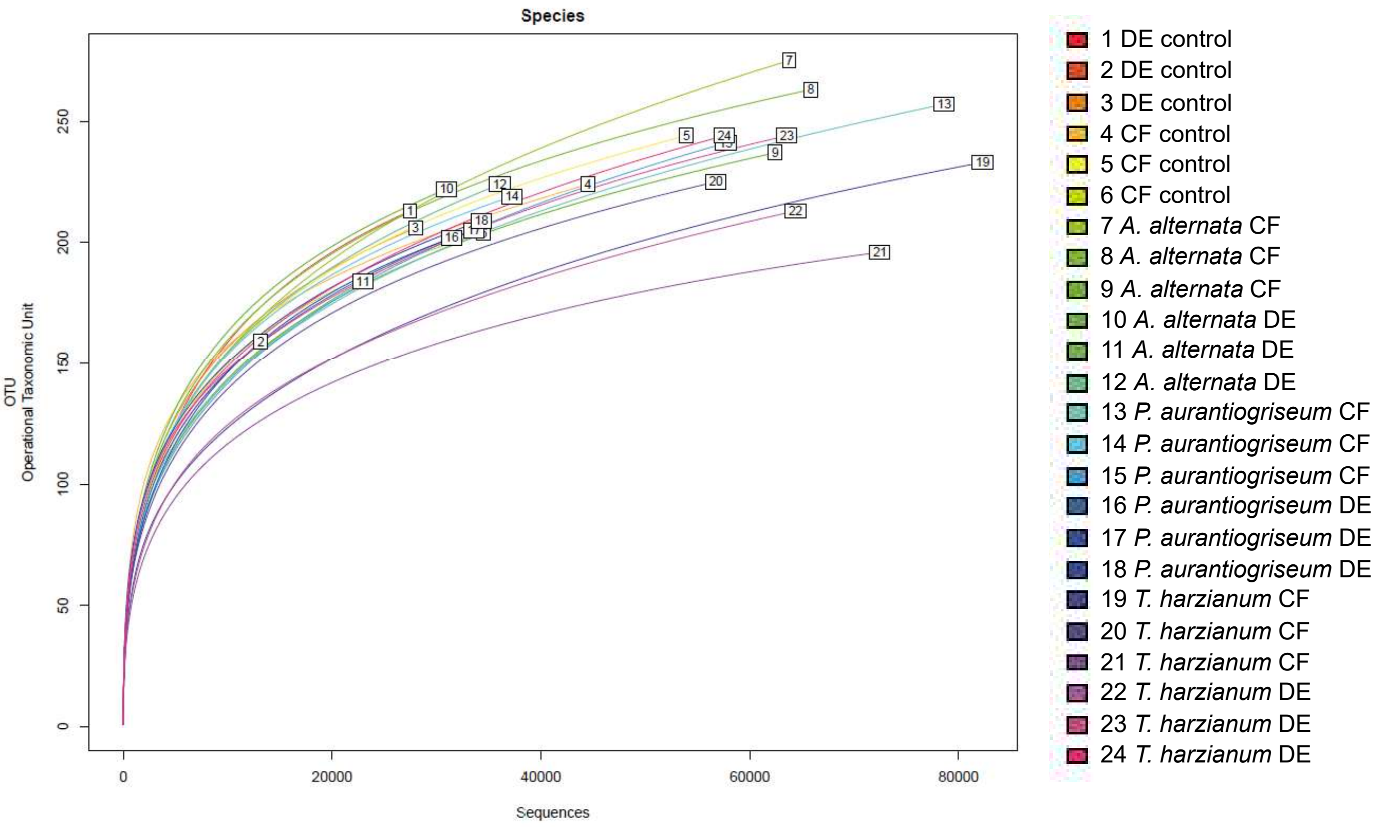

Figure S1: Rarefaction curves of (A) bacteria and (B) fungi in CF- and DE- treated and non-treated soils.

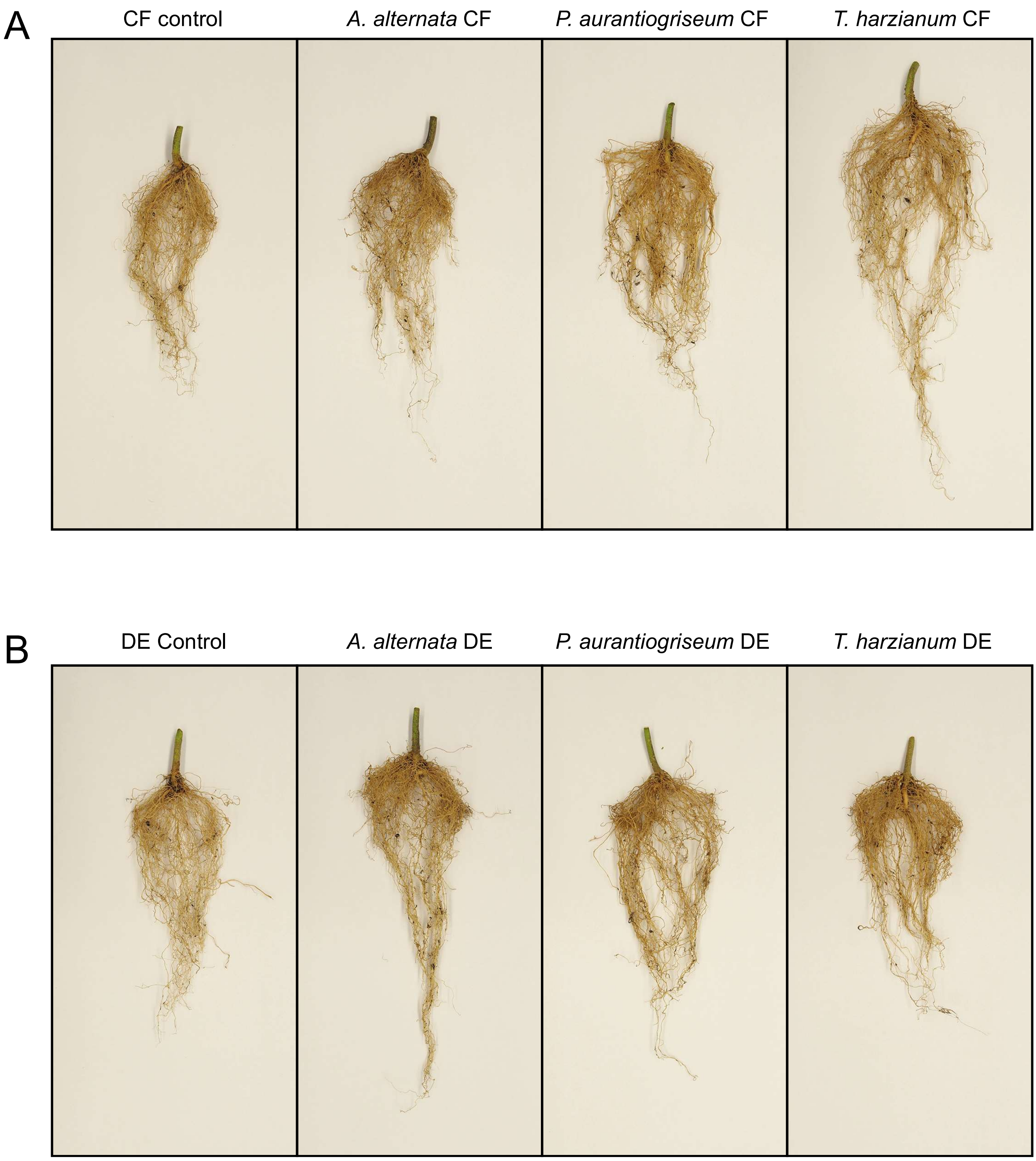

Figure S2: Soil application of fungal CFs and DEs enhances root growth. Photographs of roots of Sweet Italian pepper plants cultured with or without soil application of *A. alternata, P. aurantiogriseum* and *T. harzianum* CFs (A) or DEs (B). Photographs were taken 28 days after the first application of fungal CF.

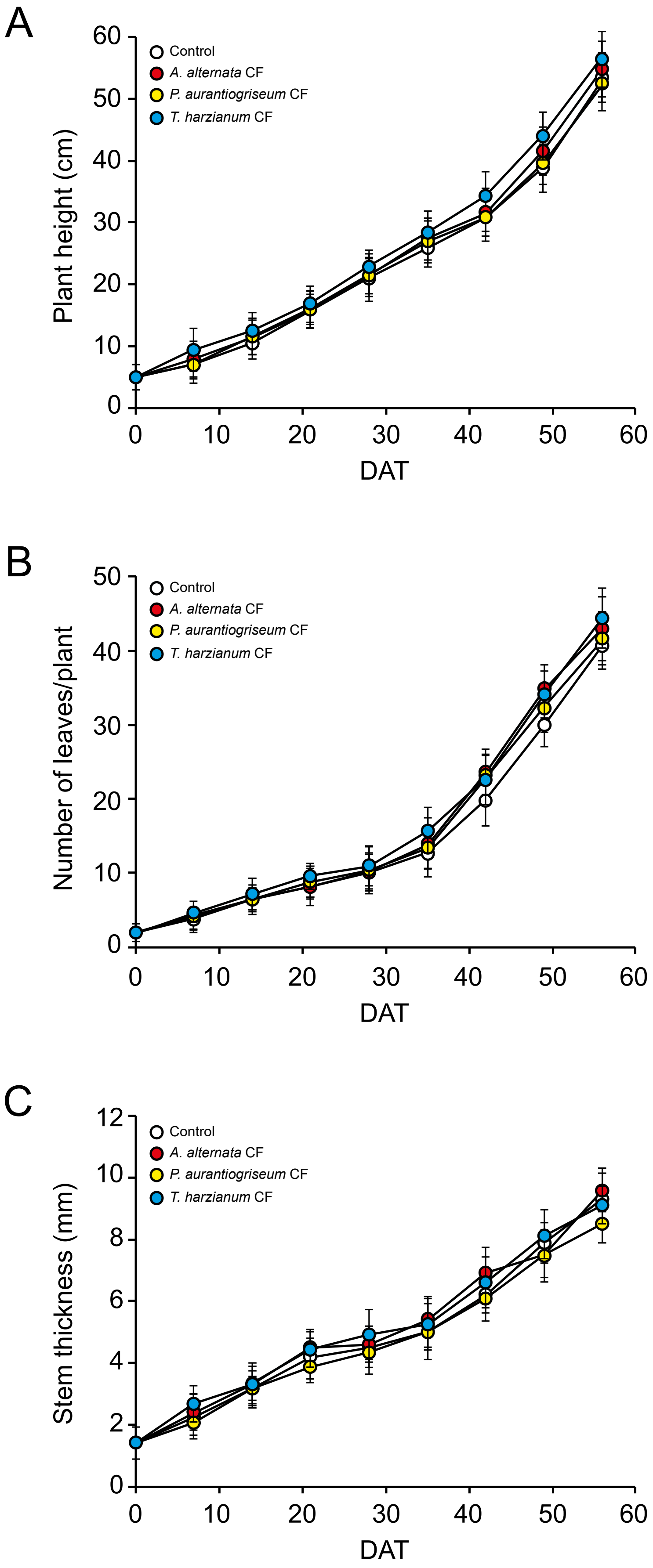

Figure S3: Soil application of fungal CFs does not promote growth of the aerial part of pepper plants. Data over time of (A) plant height, (B) number of leaves and (C) stem thickness of Sweet Italian pepper plants after the first soil application of *A. alternata, P. aurantiogriseum* and *T. harzianum* CFs. Plants were grown under greenhouse conditions. Values were obtained from two independent experiments and represent the mean ± SD of 12 different plants.

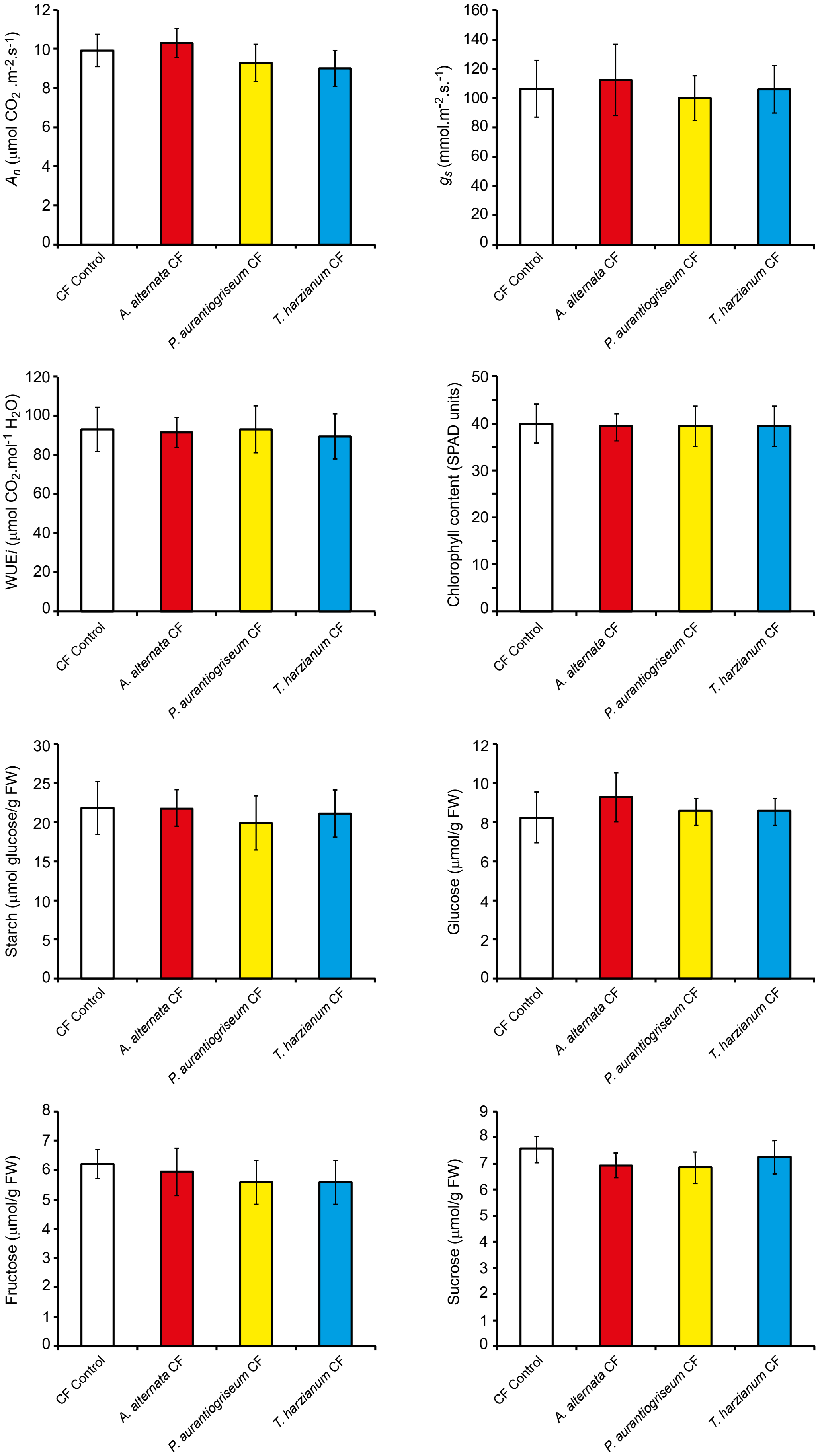

Figure S4. Soil application of fungal CFs does not promote photosynthesis and changes in the primary carbon metabolism in leaves of exposed pepper plants. A_n, g_s, WUE*i* and levels of chlorophyll and primary photosynthates (starch, glucose, fructose and sucrose) in leaves of Sweet Italian pepper plants cultured under greenhouse conditions without or with *A. alternata, P. aurantiogriseum* and *T. harzianum* CF irrigation. Values were obtained 42 days after the first treatment and represent the mean ± SD of 12 different plants.

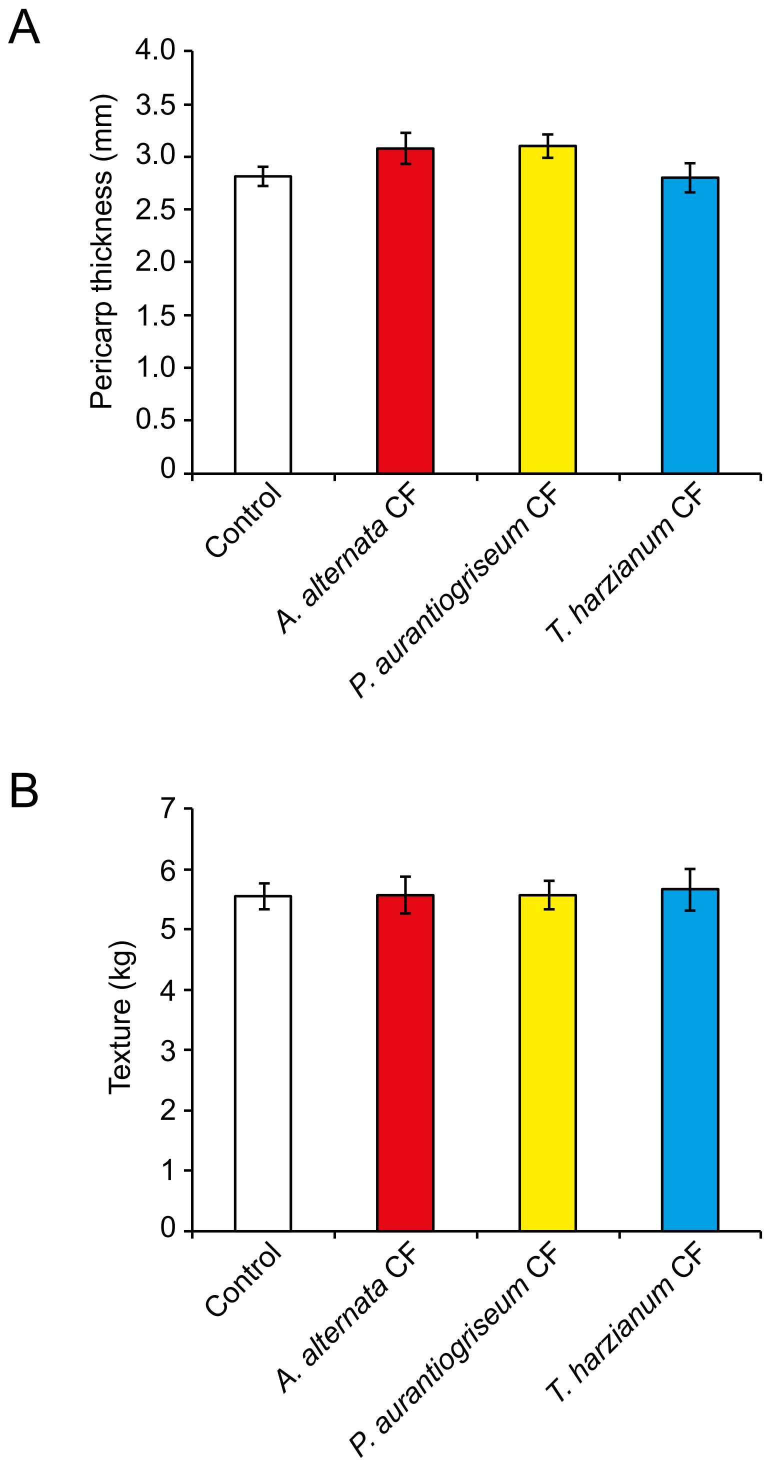

Figure S5. **Soil application of fungal CFs does not alter the thickness and texture of fruit of the treated plants.** Pericarp thickness and texture of fruits of Piquillo pepper plants cultured under open field conditions without or with *A. alternata, P. aurantiogriseum* and *T. harzianum* CF irrigation. Values were obtained 70 days after the first treatment and represent the mean ± SD of 90 fruits per treatment.

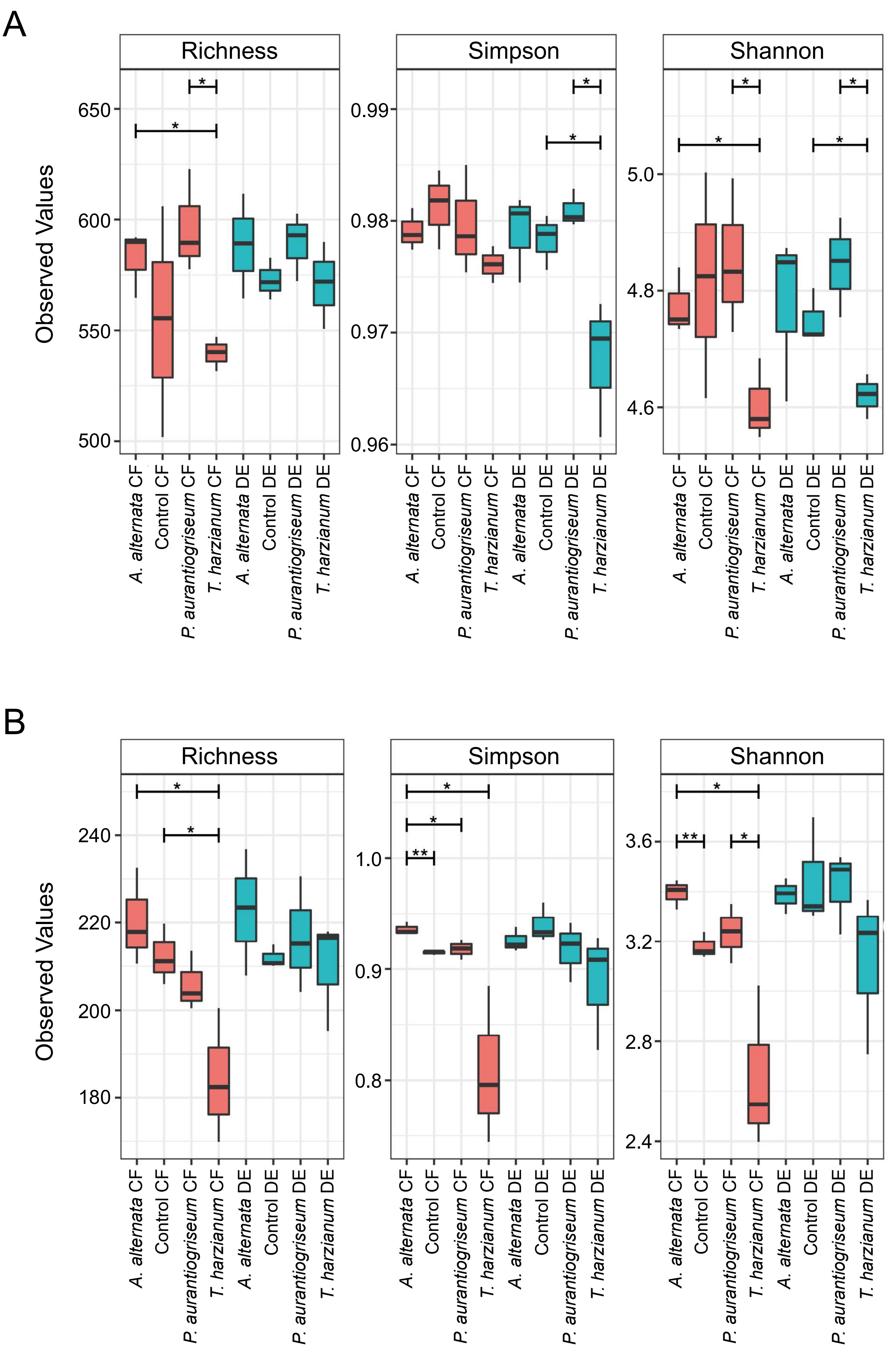

Figure S6: Microbial α diversity in soil. Richness, Simpson and Shannon indices of the bacterial and fungal populations (A and B, respectively) in soils treated with CFs and DEs of *A. alternata* (CF-AA and DE-AA, respectively), *P. aurantiogriseum* (CF-PA and DE-PA, respectively) and *T. harzianum* (CF-TH and DE-TH, respectively) and control soils.
