# Supplementary material for: Enhanced Yield of Pepper Plants Promoted by Soil Application of Volatiles From Cell-Free Fungal Culture Filtrates Is Associated With Activation of the Beneficial Soil Microbiota
Source: Front Plant Sci. 2021 Oct 21;12:752653. doi: 10.3389/fpls.2021.752653 (PMC8566893; doi:10.3389/fpls.2021.752653)
Supplement: Supplementary file 1 [file Data_Sheet_1.zip › Supplementary Table 1.PDF]

Table S1. Active CK and ABA contents (expressed as pmol g^{-1} DW) in leaves of pepper plants (cv. Sweet Italian) cultured under greenhouse conditions with or without drench application of fungal CFs. Values represent the mean ± SE from two independent experiments each consisting of two biological replicates corresponding to a pool of leaves from twelve plants. Asterisks indicate statistically significant difference in the CF treatments versus the controls according to a Student´s t-test (P<0.05).

		Control	+ *A. alternata* CF	+ *P. aurantiogriseum* CF	+ *T. harzianum* CF
CKs	tZ	2.6 ± 0.2	2.6 ± 0.8	2.3 ± 0.2	3.2 ± 0.4*
	cZ	1.8 ± 1.1	2.5 ± 0.5	3.9 ± 0.3*	2.7 ± 0.6
ABA		232 ± 12	293 ± 6*	207 ± 8	222 ± 11
