# Supplementary material for: Enhanced Yield of Pepper Plants Promoted by Soil Application of Volatiles From Cell-Free Fungal Culture Filtrates Is Associated With Activation of the Beneficial Soil Microbiota
Source: Front Plant Sci. 2021 Oct 21;12:752653. doi: 10.3389/fpls.2021.752653 (PMC8566893; doi:10.3389/fpls.2021.752653)
Supplement: Supplementary file 1 [file Data_Sheet_1.zip › Supplementary Table 2.PDF]

Table S2. Content of free amino acids (expressed as µmol/L) in *A. alternata, P. aurantiogriseum* and *T. harzianum* CFs. The results are the means ± SE of three different CFs. n.d.: not detected. Letters indicate significantly different values in a row (Student´s t-test, P>0.005)

	A. alternata CF	*P. aurantiogriseum* CF	*T. harzianum* CF
Asp	3.9 ± 0.1 b	5.2 ± 0.1 a	1.4 ± 0.1 c
Glu	4.0 ± 0.1 a	4.9 ± 0.1 a	1.3 ± 0.1 b
Ser	2.8 ± 0.1 a	3.0 ± 0.1 a	1.0 ± 0.05 b
Asn	0.17 ± 0.01 a	0.10 ± 0.03 ab	0.08 ± 0.01 b
Gly	4.0 ± 0.08 a	4.8 ± 0.5 ab	1.95 ± 0.03 b
Gln	n.d.	n.d.	1.2 ± 0.1
His	3.3 ± 0.1 a	3.3 ± 0.1 a	0.80 ± 0.00 b
Thr	6.0 ± 0.2 b	7.3 ± 0.1 c	2.4 ± 0.1 a
Ala	51 ± 1 c	182 ± 1 a	67 ± 1 b
Arg	3.9 ± 0.1 b	4.2 ± 0.1 a	1.3 ± 0.1 c
GABA	5.7 ± 0.1 b	9.5 ± 0.1 a	4.2 ± 0.1 c
Pro	4.2 ± 0.1 b	4.7 ± 0.1 a	3.4 ± 0.1 c
Tyr	5.7 ± 0.1 b	6.0 ± 0.1 a	2.9 ± 0.1 c
Val	2.1 ± 0.4 ab	2.1 ± 0.1 a	0.69 ± 0.02 b
Met	2.6 ± 0.1 a	2.6 ± 0.1 a	0.91 ± 0.1 b
Ile	3.0 ± 0.1 a	2.7 ± 0.1 b	0.93 ± 0.1 c
Leu	3.1 ± 0.1 a	2.9 ± 0.1 a	1.1 ± 0.1 b
Phe	3.9 ± 0.1 a	3.9 ± 0.1 a	1.1 ± 0.0 b
