# Supplementary material for: Enhanced Yield of Pepper Plants Promoted by Soil Application of Volatiles From Cell-Free Fungal Culture Filtrates Is Associated With Activation of the Beneficial Soil Microbiota
Source: Front Plant Sci. 2021 Oct 21;12:752653. doi: 10.3389/fpls.2021.752653 (PMC8566893; doi:10.3389/fpls.2021.752653)
Supplement: Supplementary file 1 [file Data_Sheet_1.zip › Supplementary Table 3.PDF]

Table S3. Content of CK (expressed as pmol/L) in *A. alternata, P. aurantiogriseum* and *T. harzianum* CFs. The results are the mean ± SE of three different CFs. n.d.: not detected. Letters indicate significantly different values in a row (Student´s t-test, P>0.005). CKs are classified according to their activities in plants.

		A. alternata CF	*P. aurantiogriseum* CF	*T. harzianum* CF
Precursors	iPRMP	n.d.	4.2 ± 1.3 a	1.7 ± 0.4 b
	cZRMP	28.9 ± 7.7 c	807 ± 206 a	369 ± 86 b
	∑	28.9 c	811 a	371 b
Transport forms	iPR	453 ± 17 a	66 ± 2 b	0.89 ± 0.23 c
	tZR	17.9 ± 3.2 a	10.8 ± 0.4 a	0.13 ± 0.02 b
	DHZR	4.1 ± 0.4 a	3.4 ± 0.2 a	0.16 ± 0.05 b
	cZR	349 ± 46 a	249 ± 5.7 b	0.52 ± 0.14 c
	∑	824 a	330 b	1.7 c
Active forms	iP	355 ± 106 b	6484 ± 1648 a	149 ± 15 c
	tZ	21.2 ± 5.8 c	1304 ± 241 a	162 ± 36 b
	DHZ	7.7 ± 2.1 a	7.9 ± 1.1 a	2.3 ± 0.2 b
	cZ	343 ± 79 c	55161 ± 9400 a	2008 ± 569 b
	∑	727 c	62957 a	2323 b
