# Supplementary material for: Enhanced Yield of Pepper Plants Promoted by Soil Application of Volatiles From Cell-Free Fungal Culture Filtrates Is Associated With Activation of the Beneficial Soil Microbiota
Source: Front Plant Sci. 2021 Oct 21;12:752653. doi: 10.3389/fpls.2021.752653 (PMC8566893; doi:10.3389/fpls.2021.752653)
Supplement: Supplementary file 1 [file Data_Sheet_1.zip › Supplementary Table 6.PDF]

Table S6. Bacterial and fungal abundances (expressed as ng 16S rRNA and ng ITS per µl of soil extract, respectively) in CF- and DE-treated and non-treated soils.

	Bacterial abundance	Fungal abundance
CF control	201 ± 29	205 ± 6
+ *A. alternata* CF	191 ± 22	215 ± 11
+ *P. aurantiogriseum* CF	191 ± 18	252 ± 3
+ *T. harzianum* CF	195 ± 27	219 ± 11
DE control	211 ± 22	206 ± 30
+ *A. alternata* DE	227 ± 21	181 ± 9
+ *P. aurantiogriseum* DE	199 ± 34	184 ± 5
+ *T. harzianum* DE	235 ± 7	232 ± 7
